# Immunostimulatory nanomedicines synergize with checkpoint blockade immunotherapy to eradicate colorectal tumors

**DOI:** 10.1038/s41467-019-09221-x

**Published:** 2019-04-23

**Authors:** Xiaopin Duan, Christina Chan, Wenbo Han, Nining Guo, Ralph R. Weichselbaum, Wenbin Lin

**Affiliations:** 1 Department of Chemistry, The University of Chicago, 929 E 57th St, Chicago, IL 60637 USA; 2 Department of Radiation and Cellular Oncology and Ludwig Center for Metastasis Research, The University of Chicago, 5758, S Maryland Ave, Chicago, IL 60637 USA; 3 Present Address: Cancer Research Institute, Guangdong Provincial Key Laboratory of Cancer Immunotherapy, School of Basic Medical Sciences, Southern Medical University, 510515 Guangzhou, PR China

**Keywords:** Nanoparticles, Drug delivery, Cancer microenvironment, Cancer therapy, Nanobiotechnology

## Abstract

Nanoparticles can potentially stimulate tumour microenvironments to elicit antitumour immunity. Herein, we demonstrate effective immunotherapy of colorectal cancer via systemic delivery of an immunostimulatory chemotherapeutic combination in nanoscale coordination polymer (NCP) core-shell particles. Oxaliplatin and dihydroartemesinin have contrasting physicochemical properties but strong synergy in reactive oxygen species (ROS) generation and anticancer activity. The combined ROS generation is harnessed for immune activation to synergize with an anti-PD-L1 antibody for the treatment of murine colorectal cancer tumours. The favourable biodistribution and tumour uptake of NCPs and the absence of peripheral neuropathy allow for repeated dosing to afford 100% tumour eradication. The involvement of innate and adaptive immune systems elicit strong and long lasting antitumour immunity which prevents tumour formation when cured mice are challenged with cancer cells. The intrinsically biodegradable, well tolerated, and systemically available immunostimulatory NCP promises to enter clinical testing as an immunotherapy against colorectal cancer.

## Abstract (web summary)

Nanoparticles can stimulate tumour microenvironment to promote anti-tumour immunity. Here, the authors show that the combination of delivering drugs by a nanoscale coordination polymer and anti-PD-L1 is synergistic in treating murine colorectal cancer.

## Introduction

Colorectal cancer (CRC) is the second leading cause of cancer-related deaths in the US, with an approximate lifetime risk of 1 in 20 people^1^. The standard therapy of surgery plus adjuvant chemotherapies is often limited by the side effects of and resistance to chemotherapies^2,3^. Great emphasis has thus been placed on developing immunotherapies for CRC treatment^4,5^, particularly after the Food and Drug Administration’s approval of the cytotoxic T-lymphocyte-associated protein 4 (CTLA-4) antibody ipilimumab in 2011^6^, the programmed cell death protein 1 (PD-1) antibodies pembrolizumab and nivolumab in 2014^7^, and the PD-1 ligand (PD-L1) antibody atezolizumab in 2015^8^. Clinical trials of immune checkpoint inhibitors (α-CTLA-4, α-PD-1, α-PD-L1) have shown efficacy against many cancers, but limited effect in CRCs. A small subset of CRC patients having tumours with inherently high CD8^+^ T-cell infiltration and regulatory immune checkpoint overexpression have benefitted from α-PD-1 checkpoint blockade immunotherapy^9–12^. However, this cancer phenotype represents <5% of advanced stage CRC^13,14^. The PD-L1 antibody atezolizumab showed poor response in the predominant microsatellite stable form of CRC as a monotherapy, but improved overall response rates in combination with an MEK inhibitor or α-VEGF and standard folinic acid, 5-fluorouracil, and oxaliplatin (FOLFOX) chemotherapy^15^. There is thus an established need for therapies that can improve tumour immunogenicity and induce CD8^+^ T-cell infiltration to enhance immunotherapy for the broader population of CRC patients.

Most chemotherapy regimens are considered immunologically silent or even tolerogenic. However, a subset of chemotherapeutics have recently been shown to be proinflammatory and capable of inducing immunogenic cell death (ICD), suggesting their potential for combination with checkpoint blockade to afford antitumour immunity. A key component of the FOLFOX regimen, oxaliplatin (OxPt), was identified as an ICD inducer^16–19^. We have recently shown that OxPt can be combined with a photosensitizer to synergize the ICD of OxPt-based chemotherapy and photodynamic therapy (PDT) and prime the tumour microenvironment for α-PD-L1 therapy^20^. However, PDT is a localized therapy and light penetration is limited to superficial tumours, severely limiting the clinical translation of PDT-based combination therapies in immunotherapy of CRC.

As reactive oxygen species (ROS) are primarily responsible for cancer cell death by PDT, we posited that ROS-based chemotherapeutics could induce strong ICD using chemicals instead of photons for effective priming of the tumour microenvironment. We report here that the ROS-producing drug, dihydroartemisinin (DHA), can efficiently induce ICD and exhibits synergy with OxPt. DHA is an active metabolite of artemisinin derivatives, an antimalarial drug well tolerated by millions of patients^21^. DHA contains an endoperoxide bridge that reacts with a ferrous iron catalyst to generate free radicals and cause oxidative stress, similar to the effects of PDT. Malignant cells alter iron metabolism to increase uptake and decrease efflux for tumour growth, leading to an increased pool of labile iron and thus tumour-specific activity of DHA. Despite significant potential as an anticancer therapeutic, DHA’s power has not been harnessed for in vivo antitumour treatment due to its instability in aqueous media and low bioavailability. The endoperoxide moiety that endows its antimalarial and anticancer activity reacts nonspecifically, leading to premature deactivation in circulation.

Herein, we developed self-assembled nanoscale coordination polymer (NCP) core-shell nanoparticles carrying OxPt in the core and DHA in the shell (OxPt/DHA) for their selective delivery to CRC tumours. In OxPt/DHA core-shell particles, an NCP of Zn and OxPt prodrug was coated with a lipid bilayer containing a cholesterol-DHA conjugate (chol-DHA). OxPt/DHA particles sequester the drugs from water, reductants, and proteins, enabling spatiotemporal control of drug releases in tumours and reducing systemic drug exposure. OxPt/DHA particles have desired surface properties to minimize uptake by the mononuclear phagocyte system (MPS), allowing for their selective accumulation in tumours after systemic injection. Comprised of two ICD-inducing therapeutics, OxPt/DHA elicits strong antitumour immunity in addition to anticancer efficacy, evidenced by early cell-surface exposure of calreticulin (CRT) and high mobility group box 1 (HMGB-1) protein release. Fragments of dead cancer cells were uptaken by phagocytes, leading to T-cell priming and antitumour vaccination. When supplemented with α-PD-L1, OxPt/DHA treatment completely eradicates CRC tumours, in addition to generating long-term tumour-specific immune memory response to prevent the formation and growth of new CRC tumours in mouse models.

## Results

### NCPs codeliver and stabilize synergistic OxPt and DHA

We probed the synergy between the chemotherapeutic combination of OxPt and DHA at varying ratios on two murine CRC cell lines, CT26 and MC38. As shown in Supplementary Table 1, combining OxPt with DHA led to significant reduction of the OxPt IC_50_ values (from 9.1 ± 0.7 to 1.1–3.2 μM and 10.1 ± 1.1 to 1.1–3.6 μM on CT26 and MC38 cells, respectively).

**Fig. 1:**
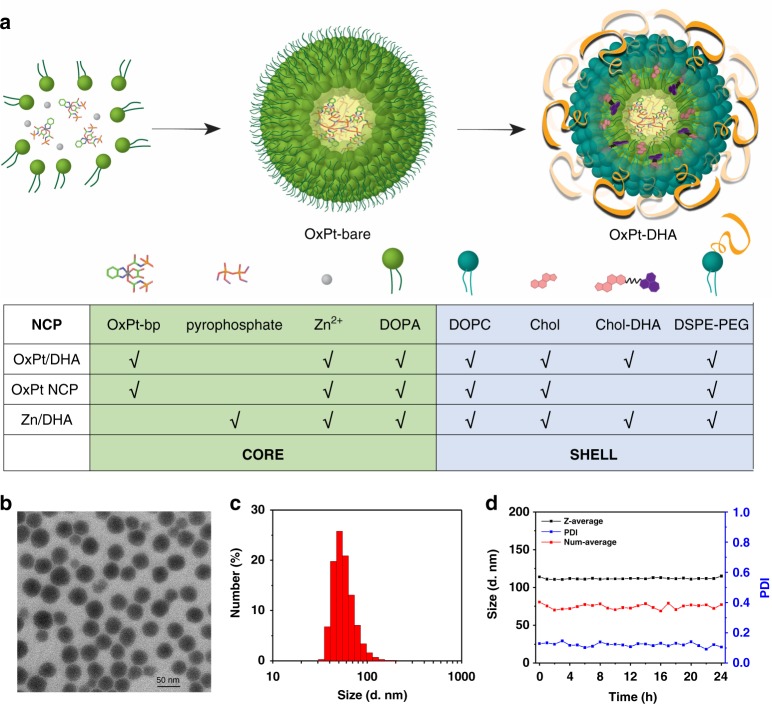
Preparation and characterization of OxPt/DHA. **a** Schematic illustration showing layer-by-layer construction of the hybrid core-shell structure of OxPt/DHA. The OxPt/DHA consists of an OxPt prodrug coordinated to Zn^2+^ ions in the core and chol-DHA in the lipid shell. Compositions of the three NCPs investigated are also shown. **b** TEM image of OxPt/DHA. **c** Number-average diameter of OxPt/DHA characterized by DLS. **d** Stability test of OxPt/DHA at 37 °C in the presence of BSA (5 mg/mL). OxPt oxaliplatin, DHA dihydroartemisinin, TEM transmission electron microscopy, DLS dynamic light scattering

To fix the molar ratios and prevent premature decomposition, we synthesized and encapsulated OxPt and DHA prodrugs, Pt(dach)(oxalate)(bisphosphoramidic acid) (OxPt-bp) and cholest-5-en-3-ol(3b)-,2-((2-((hydroartemesinincarbonyl)oxy)ethyl)disulfanyl)ethyl carbonate (chol-DHA) in NCPs (Supplementary Methods 1, 2 and Supplementary Figure 1, 2). OxPt/DHA particles were prepared in two steps (Fig. 1a). The NCP core of OxPt (OxPt-bare) was first synthesized by polymerization between Zn^2+^ ions and the phosphate groups of OxPt-bp in the presence of 1,2-dioleoyl-sn-glycero-3-phosphate (DOPA), yielding spherical OxPt-bare particles monodispersed in organic solvents (Supplementary Figure 3 and Supplementary Table 2). OxPt-bare was then coated with a mixture of 1,2-dioleoyl-sn-glycero-3-phosphocholine (DOPC), cholesterol, 1,2-diastearoyl-sn-glycero-3-phosphoethanolamine-*N*-[amino(polyethylene glycol)2000] (DSPE-PEG2k) (molar ratio 2:1:1), and different amounts of chol-DHA to afford the core-shell nanoparticles OxPt/DHA with three different OxPt:DHA ratios (1:0.5, 1:1, and 1:2), with Z-average diameters of 73.8–103.4 nm, PDIs of 0.12–0.17, and slightly negative surface charges of −20.8 to −13.0 mV in water (Fig. 1b, c and Supplementary Table 3).

DHA was conjugated to cholesterol via a disulphide bond to facilitate incorporation into the shell of the nanoparticles. The lipid bilayer protects chol-DHA from exposure to water and reductants, limiting decomposition and systemic release by preventing hydrolysis and reduction. The nanoparticles were stable with no changes in size or PDIs at 4 °C for 1 year or 37 °C for 24 h in the presence of bovine serum albumin (BSA, Fig. 1d and Supplementary Figure 4). The monotherapy nanoparticle controls OxPt NCP and Zn/DHA prepared in the absence of chol-DHA or with pyrophosphate replacing OxPt prodrug, respectively, showed similar sizes and morphology (Fig. 1a, Supplementary Figure 5, 6 and Supplementary Table 2, 3).

**Fig. 2:**
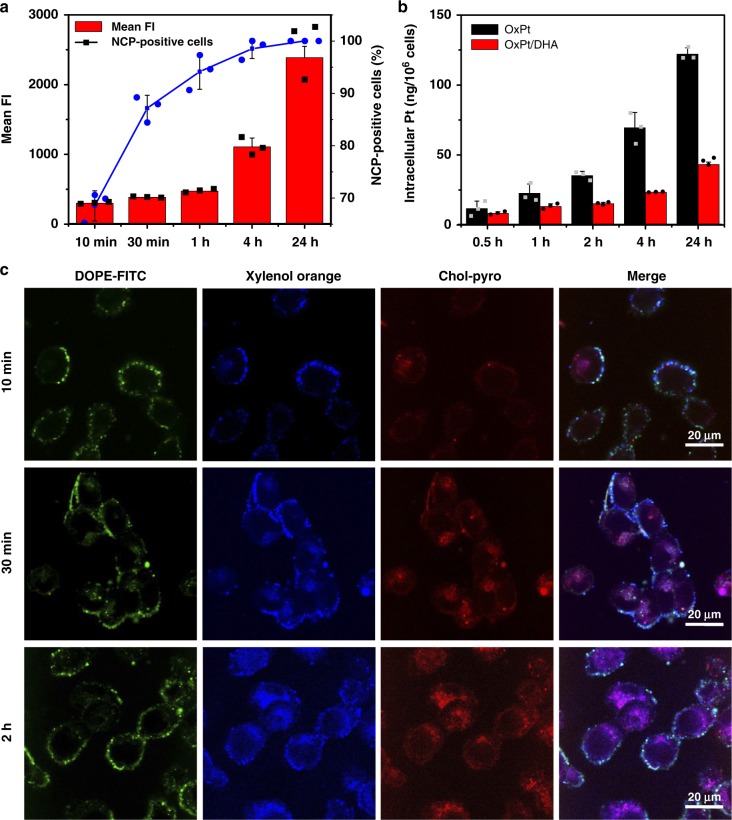
Internalization and dissociation of NCP components in cells. **a** Uptake of chol-pyro-labelled NCP particles by CT26 cells at different incubation times. **b** The intracellular Pt in CT26 incubated with OxPt or OxPt/DHA as determined by ICP-MS. **c** CT26 cells incubated with fluorescent nanoparticles containing xylenol orange in the core, chol-pyro in the shell, and FITC-DOPE lipid to visualize OxPt in the core, chol-DHA in the shell, and the lipid layer of OxPt/DHA, respectively, for different times were observed under confocal laser scanning microscopy (CLSM). The experiments were done three times and data were expressed as mean ± SD in (**a**) and (**b**). The images in (**c**) were obtained without repetition. NCP nanoscale coordination polymer, OxPt oxaliplatin, DHA dihydroartemisinin, ICP-MS inductively coupled plasma-mass spectrometry

Encapsulation into nanoparticles led to slightly higher IC_50s_ than free drugs, but showed similar trends of OxPt IC_50_ reduction when adding chol-DHA (Supplementary Table 1), suggesting that the drugs are readily released from NCPs. The synergy between the two drugs in OxPt/DHA was most readily observed with a OxPt:DHA molar ratio of 1:0.5, with a combination index of <1 at nearly all effect levels (Supplementary Figure 7). This formulation was used for all further investigations. Using OxPt/DHA particles labelled with cholesterol-conjugated pyropheophytin a (chol-pyro, Supplementary Methods 3 and Supplementary Figure 8), we showed that particles were taken up rapidly by cells, with ~95% of all cells showing particle fluorescence after 1 h. The fluorescence intensity significantly increased over time, indicating continual nanoparticle uptake (Fig. 2a). Inductively coupled plasma-mass spectrometry (ICP-MS) quantification of intracellular Pt also revealed time-dependent uptake of both nanoparticles and free OxPt. However, the uptake of nanoparticles was much less than free OxPt (Fig. 2b), possibly due to the surface PEG coating preventing interaction between particles and cells. The lower uptake explains why the particles have slightly higher IC_50_ than free drugs in vitro. To visualize the intracellular release of drugs, fluorescent nanoparticles were synthesized by doping xylenol orange (blue) into the core and coating chol-pyro (red) and FITC-DOPE (green) on the shell, which allows simultaneous visualization of OxPt in the core, chol-DHA in the shell, and the lipid layer, respectively (Supplementary Methods 4). In the first 10 min, all three dyes were primarily found on the cell surface, viewed as cyan (green and blue merged) and/or white (green, blue and red merged) in the merged image, indicating that the particles were mainly bound to the cell surface. Over time, the xylenol orange and chol-pyro nonspecifically distributed inside the cells as magenta (blue and red merged) fluorescence with varying intensity, suggesting independent release of the drugs. FITC-DOPE mainly localized to the plasma membrane (Fig. 2c), possibly due to the lipid bilayer fusion with endosome membranes to expose the NCP core and the trafficking of FITC-DOPE to the plasma membrane with the recycled endosomes. These data demonstrate that upon cellular uptake, the core−shell structure is disrupted to expose the OxPt NCP core and chol-DHA to high concentrations of intracellular reducing agents, such as glutathione (GSH, 5 mM) and ascorbate (100 μM), which further reduce and/or hydrolyse the prodrugs into parent drugs to exert efficacy.

### Triggered release of OxPt and DHA from NCPs

**Fig. 3:**
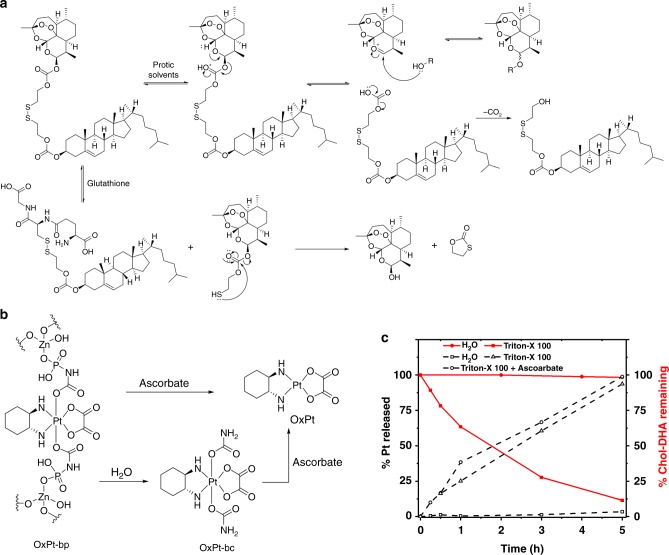
Release of DHA and OxPt from OxPt/DHA. **a** Proposed DHA release via GSH-mediated disulphide cleavage and proton-catalysed hydrolysis. **b** Proposed release of OxPt via direct reduction by ascorbate or a two-step sequence of hydrolysis to generate OxPt-bc followed by reduction to OxPt. **c** Total Pt release from and chol-DHA remaining in OxPt/DHA particle when incubated in water at 37 °C with or without 0.5% Triton X-100 and 5 mM ascorbate. The data were obtained without repetition. OxPt oxaliplatin, DHA dihydroartemisinin, GSH glutathione

The disulphide linkage of chol-DHA was cleaved by GSH at a physiologically relevant concentration to release DHA (Fig. 3a, Supplementary Figure 9). However, the kinetics of DHA release in the 5 mM GSH solution of phosphate-buffered saline (PBS) was faster than that of GSH reduction alone. Although chol-DHA was stable in aprotic, organic solvents such as THF at 37 °C for >24 h, it rapidly decomposed (>80%) in aqueous or protic solvents (Fig. 3c, Supplementary Figure 9). The addition of acids further accelerated the decomposition of chol-DHA (Supplementary Figure 9).

Based on these observations, we propose a DHA release mechanism via proton-catalysed hydrolysis in aqueous media. The protonation of the carbonate carbonyl oxygen atom in protic solvents can reversibly cleave the C−O bond next to DHA to form a DHA cation stabilized by the nearby oxygen and a carbonate monoester. The carbonate monoester irreversibly releases CO_2_, providing the driving force for the hydrolysis of DHA from chol-DHA. While GSH reduction of the disulphide linkage contributes to the release of DHA, hydrolysis of the chol-DHA carbonate linker is predominantly responsible for DHA release (Fig. 3a, Supplementary Figure 10a-b). Importantly, incorporation of chol-DHA into the lipid bilayer shell of OxPt/DHA prevented premature DHA release by limiting exposure to water. Incubation of OxPt/DHA in water at 37 °C for 24 h did not lead to significant loss of chol-DHA. However, disruption of the lipid bilayer of OxPt/DHA by Triton X-100 led to 90% degradation of chol-DHA in water at 37 °C within 5 h (Fig. 3c). The core−shell structure of OxPt/DHA thus protects DHA from exposure to water and reductants during circulation, ensuring the selective delivery of DHA to cancer cells.

The Pt(IV) prodrug OxPt-bp was also reduced via two mechanisms: direct reduction into OxPt or a two-step sequence of hydrolysis to generate Pt(dach)(oxalate)(biscarbamate) (OxPt-bc) followed by reduction to OxPt (Fig. 3b, Supplementary Figure 10c-d, 11). The structure of OxPt-bc was confirmed by single crystal X-ray diffraction (Supplementary Figure 12, Supplementary Table 4). Interestingly, only ascorbate and not GSH can reduce either OxPt-bp or OxPt-bc to afford OxPt (Fig. 3b, Supplementary Figure 10b). While OxPt does not release from intact OxPt/DHA particles, the disruption of the lipid bilayers upon endocytosis allows access of the robust coordination polymer to ascorbate and acidic aqueous medium, leading to release of both OxPt and OxPt-bc (Fig. 3b, c, Supplementary Figure 10c).

### Cell death by classically programmed and immunogenic pathways

**Fig. 4:**
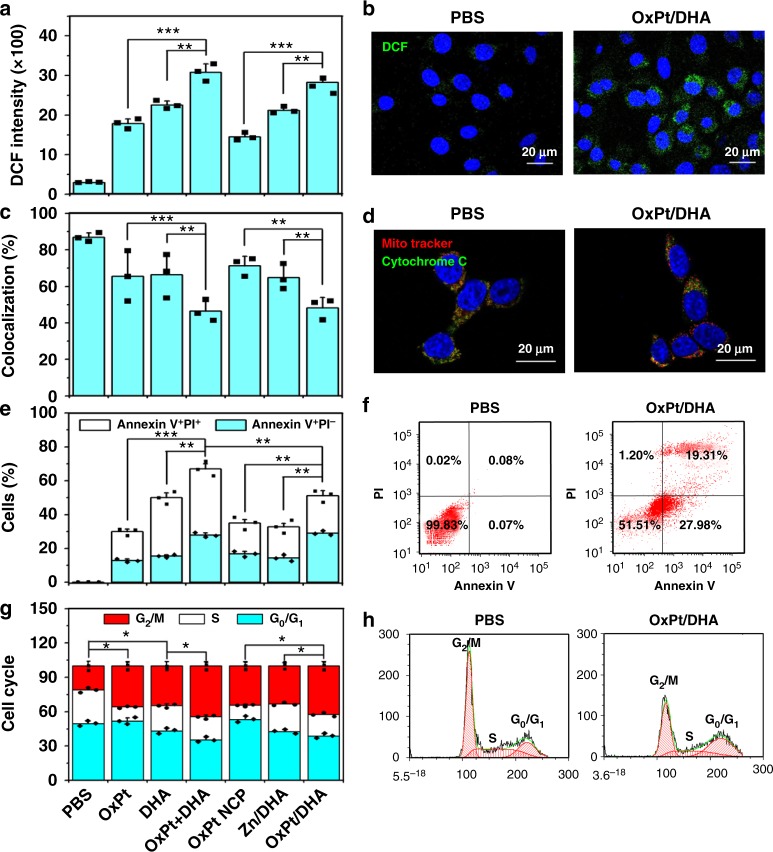
Programmed cell death in colorectal cancer cells by ROS generation. **a**, **b** ROS generation in cells treated with OxPt/DHA, as indicated by the green fluorescence of 2′,7′-dichlorofluorescein (DCF) that was oxidized from 2′,7′-dichlorodihydrofluorescein diacetate (H_2_DCFDA) by ROS. **c**, **d** Release of cytochrome *c* from mitochondria in cells incubated with OxPt/DHA. Mitochondria (red fluorescence) and cytochrome *c* (green fluorescence) were stained by MitoTracker Red CMXRos and anti-cytochrome *c* antibody, respectively. **e**, **f** Apoptosis induced by OxPt/DHA. After treatment, cells were stained by Alexa Fluor 488-labelled Annexin V and propidium iodide (PI) and analysed by flow cytometry. **g**, **h** Cell cycle arrest caused by OxPt/DHA. Treated cells were fixed with 70% ethanol overnight, treated with RNase A, stained by PI, and analysed by flow cytometry. Data are expressed as means ± SD, and one of three repetitions with similar results is shown here. *p < 0.05, **p < 0.01, ***p < 0.001 by Student’s two-tailed *t* test. OxPt oxaliplatin, DHA dihydroartemisinin, ROS reactive oxygen species

The purported antimalarial activity of DHA involves cleavage of the endoperoxide bridge by ferrous iron, generating oxygen- and carbon-based radicals. As cancer cells are highly sensitive to agents that can augment oxidative stress, we investigated whether our combination therapy can synergistically generate ROS, which can directly react with the membrane, DNA, proteins, and organelles or generate secondary products to cause damage. Both OxPt and DHA can individually induce ROS generation in tumour cells, with a significant increase when given in combination (*p* *<* 0.001; Fig. 4a, b and Supplementary Figure 13). ROS is a known trigger for dysfunction of mitochondria, which regulates both autophagy and apoptosis. The generated ROS causes release of cytochrome *c* from mitochondria, as evidenced by the decrease in the colocalization between the mitochondria (red) and the cytochrome *c* (green) fluorescence (Fig. 4c, d and Supplementary Figure 14), disrupting the membrane potential as a consequence of ROS accumulation. As a result, both OxPt and DHA induced programmed cell death by apoptosis/necrosis (Fig. 4e, f and Supplementary Figure 15). The combination of OxPt and DHA increased both early apoptotic Annexin V^+^/PI^−^ cells (26.8 ± 1.4% compared to 11.9 ± 1.0% and 14.7 ± 1.7% for OxPt and DHA, respectively) and late apoptotic/necrotic Annexin V^+^/PI^+^ cells (36.2 ± 3.0% compared to 15.6 ± 1.5% and 31.6 ± 2.9% for OxPt and DHA, respectively). Treatment with OxPt NCP, Zn/DHA, and OxPt/DHA led to similar trends in the ROS, cytochrome *c* release, and induction of apoptosis (Fig. 4a−f and Supplementary Figure 13-15).

In addition to mitochondrial dysfunction, ROS can also inhibit cell growth by cell cycle arrest via endoplasmic reticulum (ER) stress. G_2_/M phase cell cycle arrest was observed in CT26 cells treated by either OxPt or DHA, increasing the percentages of cells in the G_2_/M phase to 35.6 ± 3.7% (*p* = 1.1 × 10^−2^) and 34.5 ± 3.9% (*p* = 1.5 × 10^−2^), respectively, from 20.8 ± 4.4% in PBS. Combining OxPt and DHA further increased the proportion of cells arrested at the G_2_/M phase to 44.3 ± 3.7% (*p* = 4.4 × 10^−2^ and 3.4 × 10^−2^ compared to OxPt and DHA, respectively). Two sequential, redundant mechanisms for ER stress-regulation of the cell cycle have been suggested: first delay at the G_2_ checkpoint followed by cell cycle arrest at the G_1_ checkpoint^22^. Interestingly, treatment with OxPt specifically decreased the accumulation of S phase cells, whereas treatment with DHA led to approximately equal reductions of cells in the G_0_/G_1_ and S phases. Cells treated with OxPt NCP, Zn/DHA, and OxPt/DHA resulted in similar changes in the cell cycle as free drug treatments (Fig. 4g, h and Supplementary Figure 16).

**Fig. 5:**
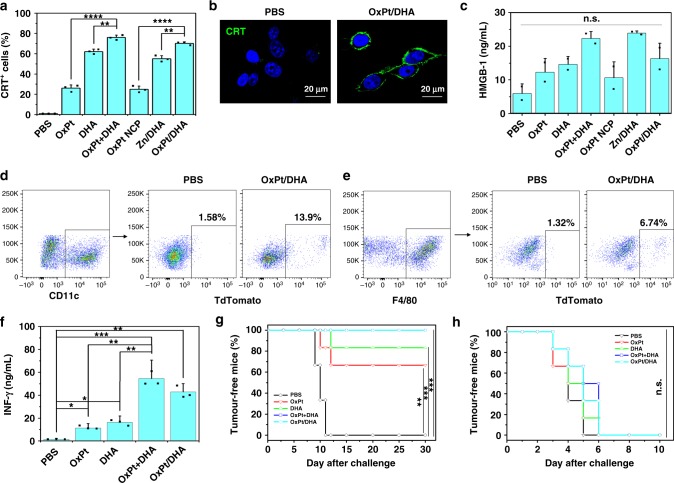
Immunostimulatory effects in colorectal cancer cells. **a**, **b** CRT cell surface expression upon treatment with OxPt/DHA, determined by flow cytometry (**a**) and CLSM (**b**). **c** HMGB-1 release from tumour cells treated with OxPt/DHA, detected by ELISA. **d**–**f** Uptake of treated MC38 cells by bone-marrow-derived dendritic cells (**d**) and macrophages (**e**). **f** Priming of T-cell responses triggered by OxPt/DHA. MC38 tumour cells were treated with OxPt/DHA, and injected into the right footpads of C57BL/6 mice to determine the capacity of draining lymph node cells to produce IFN-γ in response to MC38 lysates. **g**, **h** In vivo anticancer vaccination of OxPt/DHA in immunocompetent C57BL/6 mice (**g**) and immunodeficient Rag^2−/−^ mice (**h**). The antitumour response was measured by immunizing mice with OxPt/DHA-treated tumour cells in one flank and challenging mice with untreated, live tumour cells in the opposite flank 7 days later. Data are expressed as means ± SD. One of three repetitions with similar results is shown here for (**a**)−(**e**). The result was obtained without repetition for (**f**)−(**h**) (*n* = 6). *p < 0.05, **p < 0.01, ***p < 0.001, ****p < 0.0001 by Student’s two-tailed *t* test. CRT calreticulin, OxPt oxaliplatin, DHA dihydroartemisinin, CLSM confocal laser scanning microscopy

ER stress and ROS production are essential components of the intracellular pathways that govern ICD, which occur in parallel to activate danger signalling pathways that help to traffic damage-associated molecular patterns (DAMPs) to the extracellular space^16,23–26^. We confirmed that OxPt is able to induce ICD, and demonstrated that DHA can also effectively induce ICD, as evidenced by calreticulin (CRT) cell-surface exposure (Fig. 5a, b, Supplementary Figure 17). We further quantified the release of high mobility group box-1 (HMGB-1) from cells treated with both drugs by enzyme-linked immunosorbent assay (ELISA). Incubation with OxPt or DHA led to increased HMGB-1 release from cells, which was further increased by coincubation with both drugs (Fig. 5c).

### Priming a CRC tumour-specific immune response for efficacy

OxPt- and/or DHA-treated tdTomato-transfected MC38 cells could be engulfed by bone-marrow-derived dendritic cells (DCs) and macrophages (Fig. 5d, e and Supplementary Figure 18-20). Using tdTomato-MC38-OVA cells, we showed that treatment with OxPt/DHA resulted in significantly higher cross-presentation of the ovalbumin (OVA) peptide onto MHC I, as demonstrated by staining of the SIINFEKL-H2kb complex on the surfaces of DCs and macrophages (Supplementary Figure 21, 22). This result suggests that both phagocytes are involved in presenting tumour antigens to initiate the adaptive immune response^27^.

To investigate whether OxPt/DHA could prime T cells, dead and/or dying MC38 cells treated with OxPt/DHA were inoculated into the footpads of C57BL/6 mice. Six days after inoculation, the regional popliteal lymph nodes were excised and stimulated with MC38 lysates ex vivo. Both OxPt- and DHA-treated cells were able to prime T cells for IFN-γ production (Fig. 5f), with the combination of OxPt and DHA showing the highest ability to prime T cells. In addition, the T cell priming ability of OxPt/DHA-treated MC38 cell lysates was much stronger than that of the known MC38 antigen KSPWFTTL (Supplementary Figure 23).

Activation of T cells by OxPt and/or DHA treatment led to efficient vaccination specifically against MC38. OxPt- or DHA-treated cells reduced the frequency of tumours developing from live cells to 33 and 17%, respectively, by day 30 (Fig. 5g). In comparison, 100% mice developed tumours with PBS-treated cells. This is consistent with in vitro results showing DHA is a stronger ICD inducer than OxPt, with a greater percentage of CRT^+^ cells and more HMGB-1 secretion. No tumour formation occurred when live MC38 cells were inoculated into mice vaccinated with OxPt+DHA- or OxPt/DHA-treated cells, but the immune system did not recognize the unrelated Lewis lung carcinoma LL/2 cells, leading to 100% tumour formation (Supplementary Figure 24). Furthermore, these protective immune responses were lost in immunodeficient Rag^2−/−^ mice, leading to 100% tumour formation in mice regardless of vaccination (Fig. 5h and Supplementary Figure 25).

**Fig. 6:**
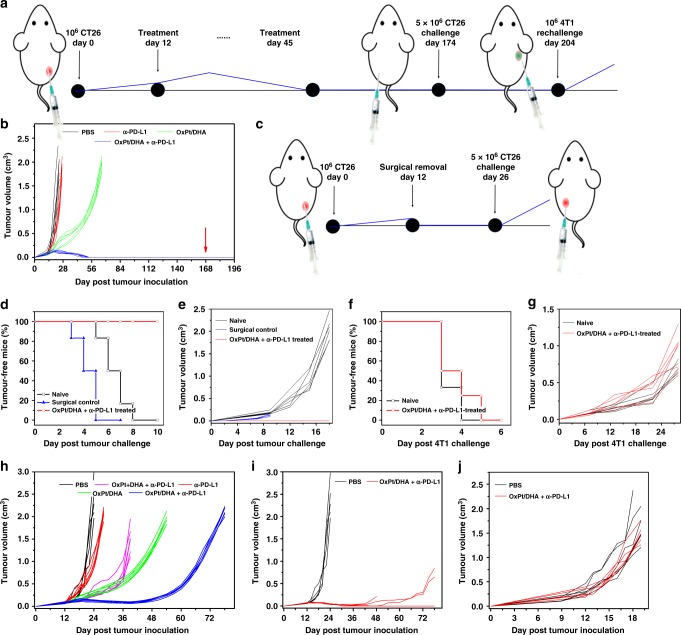
Enhanced anti-PD-L1 immunotherapy on colorectal cancers. **a** Experimental design for the treatment and challenge of CT26 tumour-bearing mice. Tumours were allowed to grow for 12 days before treatment to form more immunosuppressive tumours. Then, tumour-bearing mice were intraperitoneally injected with OxPt/DHA combined with α-PD-L1 every 3 days for 12 total doses. Three months after all tumours had disappeared, mice were challenged with CT26 cells, followed by rechallenge with 4T1 1 month later. **b** Growth curves of CT26 tumours after treatment with OxPt/DHA combined with α-PD-L1 and challenge with CT26 cells (red arrow). **c** Experimental design for surgery control of CT26 tumour-bearing mice. Percentage tumour-free mice (**d**, **f**) and tumour growth curve (**e**, **g**) after challenge with CT26 cells (**d**, **e**) or rechallenge with unrelated 4T1 tumour cells (**f**, **g**) in naïve mice or OxPt/DHA and α-PD-L1-treated mice. **h** Growth curves of MC38 tumour on C57BL/6 after treatment with OxPt/DHA (8 mg/kg OxPt) combined with α-PD-L1. **i**, **j** Therapeutic effect of OxPt/DHA plus α-PD-L1 on C57BL/6 (**i**) at the dose of 16 mg/kg OxPt and Rag2^−/−^ mice (**j**) at the dose of 8 mg/kg OxPt. Data are each pooled from two independent experiments and expressed as means ± SD (*n* = 6 except for (**j**); *n* = 5 for (**j**)). OxPt oxaliplatin, DHA dihydroartemisinin

Recognizing the immunogenicity of OxPt and DHA, we investigated OxPt/DHA in combination with α-PD-L1 checkpoint blockade. Syngeneic tumour models of CT26 and MC38 were established on immunocompetent BALB/c and C57BL/6 mice, respectively, and tumours were allowed to grow for approximately 12 days, reaching 80–120 mm^3^ before treatment. As minimal toxicity by body weight was observed after 50 doses of Zn/DHA at 5 mg DHA/kg, four weekly doses of OxPt/DHA at 60 mg OxPt/kg, or one dose of OxPt/DHA at 80 mg OxPt/kg (Supplementary Figure 26), we chose a dose of 8 mg OxPt/kg, 2.8 mg DHA/kg, and/or 75 μg α-PD-L1/mouse. This dose was at least ten times lower than the maximum tolerated dose (MTD) and allowed for frequent low-dose metronomic dosing as opposed to conventional infrequent doses at or near the MTD^28^. Mice were i.p. injected once every 3 days for up to 12 total doses (Fig. 6a). With significant OxPt accumulation in tumours 72 h post administration (Supplementary Figure 27), this dosing schedule allowed for a dose-dense chemotherapy schedule with a near continuous presence of chemotherapy in the tumour. This was in line with the Norton−Simon hypothesis that more frequent doses will lead to greater clinical benefit by minimizing the opportunities for cancer regrowth between doses^29^. Importantly, OxPt/DHA increased the effective doses of OxPt and DHA by ~15 and 1000 times, respectively (Table 1). Nearly all of the free drugs were cleared out of or decomposed in the bloodstream within 1 h of intravenous injection (Supplementary Figure 28, 29). Despite this, free OxPt leads to significant peripheral neuropathy while OxPt/DHA showed no such toxicity (Supplementary Figure 30).

**Table 1:** OxPt and DHA pharmacokinetic information

Parameter (Unit)	*t*_1/2_ alpha (h)	*t*_1/2_ beta (h)	AUC 0-inf (ID%mL^−1^ h)	AUMC (ID%mL^−1^ h^2^)	MRT (h)
Free OxPt	0.18 ± 0.02	14.67 ± 3.04	5.77 ± 2.65	122.5 ± 77.5	20.1 ± 4.4
Free DHA^a^	(0.22 ± 0.04)^b^		(0.03 ± 0.01)^b^	(0.59 ± 0.16)^b^	(0.31 ± 0.06)^b^
OxPt/DHA	0.27 ± 0.30 (0.39 ± 0.38)^b^	20.70 ± 4.50 (7.01 ± 1.13)^b^	80.9 ± 11.2 (33.6 ± 5.8)^b^	2431.5 ± 809.7 (330.2 ± 79.8)^b^	29.51 ± 6.27 (9.79 ± 1.41)^b^

Data are expressed as means ± SD

*OxPt* oxaliplatin, *DHA* dihydroartemisinin

^a^This was fitted to a one-compartment model

^b^The numbers in parentheses refer to DHA values

In CT26 tumour-bearing mice, the low-dose α-PD-L1 treatment alone was ineffective at controlling tumour growth. The combination of free OxPt, DHA, and α-PD-L1 proved moderately effective (average tumour volumes of 203.27 ± 81.00 mm^3^ on day 18 compared to 616.80 ± 46.59 mm^3^ for PBS) but extremely toxic, as the body weights steadily decreased (Supplementary Figure 31). All mice had to be euthanized after three doses for humanitarian reasons in accordance with our animal protocols. We hypothesized that NCPs can decrease the toxicity of OxPt and DHA by providing a favourable biodistribution profile. In CT26 tumour-bearing mice, <5%ID Pt/g accumulated in key organs such as the liver, spleen, and kidney indicating OxPt/DHA is not significantly uptaken by the MPS. Furthermore, there was <3%ID Pt/g accumulation in the heart and lung, suggesting OxPt/DHA does not aggregate in circulation. The low MPS uptake and slow clearance led to progressive accumulation in tumour to a maximum of 12.3 ± 2.8 %ID/g at 48 h post administration as a result of the enhanced permeability and retention effect compared to 0.56 ± 2.8%ID/g by the free drug (Supplementary Figure 27, 32). OxPt NCP with or without α-PD-L1 was well tolerated and led to similar tumour growth rates, significantly controlling tumour growth and retarding tumour growth for over 1 month. Interestingly, though Zn/DHA did not show substantial anticancer efficacy alone or in combination with α-PD-L1 (Supplementary Figure 33), it significantly enhanced the efficacy of OxPt and delayed tumour growth to ~2 cm^3^ until day 66. The tumour growth curve of OxPt/DHA plus α-PD-L1 was initially similar to that of OxPt/DHA, but started to deviate around day 18, after which all of the tumours regressed and eventually disappeared on days 40–50. No tumour recurrence was observed for a period of 120 days (Fig. 6b).

The antitumour immune response initiated by OxPt/DHA plus α-PD-L1 resulted in a memory response; OxPt/DHA plus α-PD-L1-treated mice which were tumour-free for at least 120 days were challenged with live CT26 cells on the contralateral flank. No mice grew new tumours over the next month compared to 100% tumour formation in naïve mice inoculated with the same cell passage or mice with surgically removed CT26 (Fig. 6b−e), indicating OxPt/DHA plus α-PD-L1 generated long-lasting antitumour immunity to prevent cancer relapse. The immune memory was found to be tumour specific, as subsequent rechallenge with live murine mammary adenocarcinoma 4T1 cells in the mammary fat pad showed no difference in growth compared to cells implanted in naïve mice (Fig. 6f, g).

We confirmed these results on the more immunosuppressive model of MC38 tumours in C57BL/6 mice, which yielded similar results under the same treatment regimen (Fig. 6h, Supplementary Figure 34). The chemoimmunotherapy of free OxPt, DHA, and α-PD-L1 was initially effective at controlling tumour growth, but eventually the tumours grew aggressively and the mice had to be euthanized due to the tumour burden on day 39. OxPt/DHA showed similar anticancer efficacy, but continued to control the tumours until day 54. Tumours treated with OxPt/DHA plus α-PD-L1 regressed around day 18 and were controlled for an extended period of time, but eventually grew back (Fig. 6h). By increasing the chemotherapy dose of OxPt/DHA to 16 mg OxPt/kg and 4.6 mg DHA/kg plus 75 μg α-PD-L1/mouse, three out of five treated mice were tumour free and the remaining two mice showed prolonged tumour growth control (Fig. 6i). The contribution of the immune system to OxPt/DHA plus α-PD-L1 anticancer efficacy was immediately obvious as no therapeutic effect was observed when MC38 tumours implanted in immunodeficient Rag^2−/−^ mice were treated with OxPt/DHA plus α-PD-L1 (Fig. 6j).

### Engaging the innate and adaptive immune systems

**Fig. 7:**
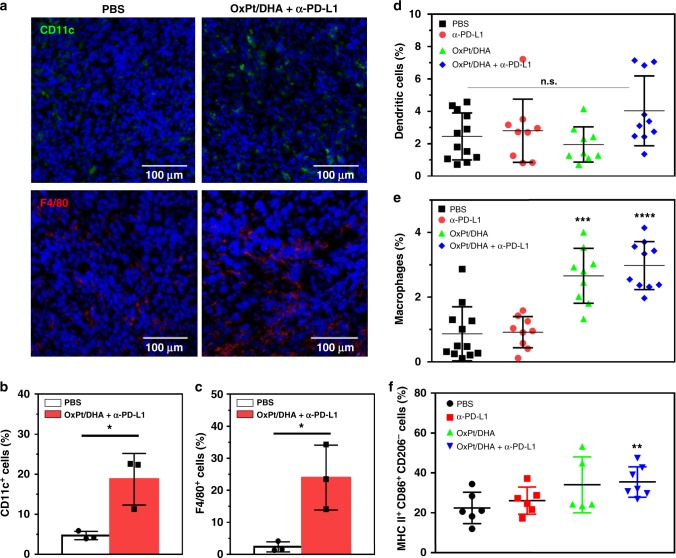
Tumour infiltration of innate immune cells. **a** Immunofluorescence analysis showing the infiltration of dendritic cells and macrophages 2 days after treatment. **b**, **c** The densities of CD11c^+^ (**b**) and F4/80^+^ (**c**) cells in the whole tumours 2 days after treatment, from the confocal images of immunofluorescence staining (*n* = 3). **d**, **e** The percentages of dendritic cells (**d**), total macrophages (**e**) and M1 macrophages (**f**) by flow cytometry of cell-surface staining 12 days after the first treatment. Data are each pooled from three independent experiments for (**d**) and (**e**). Data were obtained without repetition for (**f**). *p < 0.05, **p < 0.01, ***p < 0.001, ****p < 0.0001 by Student’s two-tailed *t* test

Immunogenic therapies are known to induce an innate immune response, including rapid infiltration of phagocytic DCs and macrophages. As the activation of DCs may constitute the first of several steps in the immune development process^30^, we first investigated the intratumoural levels of antigen-presenting innate immune cells. We observed increased tumour infiltration of CD11c^+^ and F4/80^+^ cells 2 days after the first treatment with OxPt/DHA plus α-PD-L1 (Fig. 7a−c). The main function of DCs is to process antigen materials and present them to T cells to promote immunity^31,32^, while macrophages can engulf and digest cellular debris and secrete proinflammatory cytokines to recruit other immune cells in addition to presenting antigens to DCs^33^. The enhanced infiltration of DCs and macrophages in tumours might have resulted from the improved immunogenicity caused by OxPt/DHA, allowing for efficient antigen capture and presentation. We also investigated the infiltration of DCs and macrophages 12 days after the first treatment. The percentage of DCs slightly, though not significantly, increased in tumours treated with OxPt/DHA plus α-PD-L1 (Fig. 7d). As this is also equivalent to the third day after the fourth treatment, the lower infiltration may also coincide with DC migration to the regional lymph nodes for antigen presentation^34^. The percentages of macrophages were still significantly increased in mice treated with either OxPt/DHA alone or in combination with α-PD-L1 (Fig. 7e). Only OxPt/DHA plus α-PD-L1 significantly increased the percentage of M1 macrophages in total macrophages in tumours (Fig. 7f), suggesting the combination treatment enhances polarization to M1 macrophages or recruits more M1 macrophages into tumours to facilitate antigen presentation. By recruiting DCs and macrophages to phagocytize dying and/or dead cancer cells and enhancing the processing/presentation of tumour-associated antigens to T cells, OxPt/DHA may have increased the density of CD8^+^ T cells in tumours to potentiate checkpoint blockade immunotherapy.

**Fig. 8:**
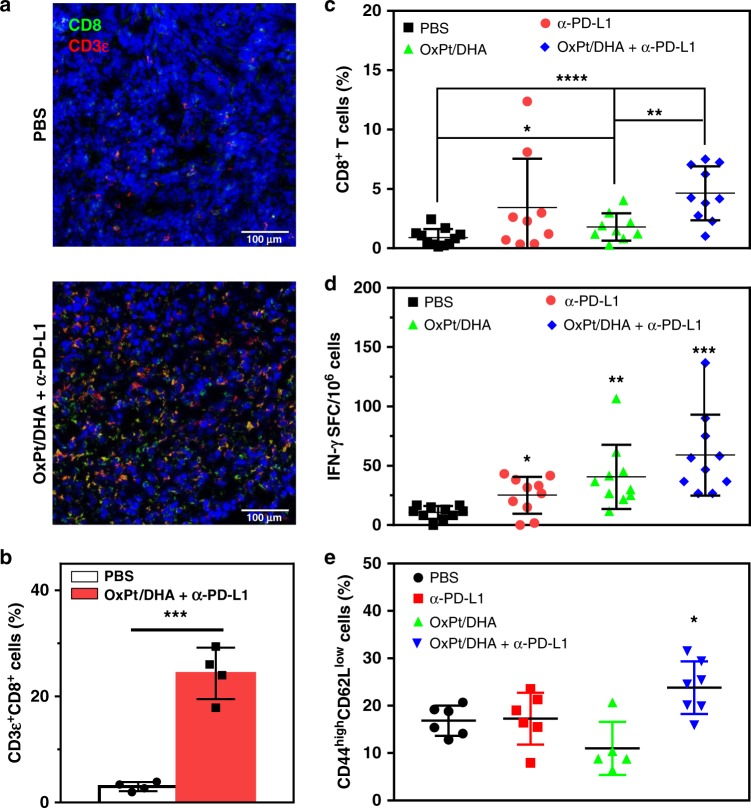
OxPt/DHA promotes tumour-specific T cell response. **a** Immunofluorescence analysis showing the infiltration of CD8^+^ T cells 12 days after the first treatment. **b** The density of CD8^+^ T cells in the tumour sections, analysed from the confocal images of immunofluorescence staining (*n* = 3). **c** CD8^+^ T cells in tumours by flow cytometry of cell-surface staining 12 days after the first treatment. **d** KSPWFTTL antigen-specific IFN-γ producing T cells detected by ELISPOT assay 12 days after the first treatment. **e** The percentage of effector memory T cells in total CD8^+^ T cells in spleens after treatment with OxPt/DHA plus α-PD-L1. Data are each pooled from two independent experiments for (**c**)−(**e**). *p < 0.05, **p < 0.01, ***p < 0.001 and ****p < 0.0001 by Student’s two-tailed *t* test. OxPt oxaliplatin, DHA dihydroartemisinin, ELISPOT Enzyme-Linked ImmunoSpot

Tumours with low densities of CD8^+^ T cells, such as MC38, do not generally respond to PD-1/PD-L1 blockade^35–37^. Effective combination therapy can increase the intratumoural infiltration of CD8^+^ T cells to significantly increase the response rate. Immunofluorescence analysis performed 12 days after the first treatment with OxPt/DHA plus α-PD-L1 showed significant increase in the density of infiltrating CD3ε^+^ cells, which were primarily CD8^+^ (Fig. 8a, b, Supplementary Figure 35,36), providing the evidence for treatment-related change in the tumour microenvironment increasing the number of cytotoxic T cells. The flow cytometry data also showed that mice treated with OxPt/DHA had an increase in the number of CD8^+^ T cells in tumours, which increased further when α-PD-L1 was added (Fig. 8c), but no significant difference was observed in total CD45^+^ leucocyte or CD4^+^ T cell infiltration (Supplementary Figure 37,38). CD8^+^ T cells increased 0.97-fold (*p* = 2.5 × 10^−2^) and 4.09-fold (*p* = 2.7 × 10^−5^) in tumours treated with OxPt/DHA alone or with α-PD-L1, compared with PBS group, respectively. The immune response was further determined to be MC38-specific by an Enzyme-Linked ImmunoSpot (ELISPOT) assay to detect the presence of tumour antigen-specific T cells in the leucocyte-abundant spleens of MC38 tumour-bearing mice (Fig. 8d). The harvested splenocytes were stimulated with KSPWFTTL, the tumour-associated antigen peptide presented by MHC I H-2K^b^, for 48 h to detect the antigen-specific CD8^+^ T cells. The number of antigen-specific IFN-γ producing T cells was significantly increased in tumour-bearing mice treated with OxPt/DHA, indicating that OxPt/DHA effectively generates tumour-specific T cell response, which could be further enhanced by the addition of α-PD-L1. Further investigation demonstrated a significant increase in CD44^high^CD62L^low^ effector memory T cells in the spleen after treatment with OxPt/DHA plus α-PD-L1 (Fig. 8e), which suggests that T cells might be involved in the long-term memory response. However, the exact subset of responsible immune cells remains to be elucidated.

## Discussion

As a well-tolerated antimalarial drug, DHA is a strong candidate for anticancer therapy. Higher levels of iron in tumours catalytically decomposes the reactive endoperoxide to generate ROS. However, the low bioavailability and rapid decomposition of DHA in sera present two major obstacles to using DHA in cancer therapy. By synthesizing a cleavable prodrug and encapsulating it into the lipid bilayer shell of an NCP, DHA can be shielded from the water in circulation and released only after particle dissociation upon cellular uptake. Intracellular release of DHA from chol-DHA can occur via disulphide cleavage and hydrolysis. The OxPt-bp coordination polymer can also release free OxPt directly by ascorbate reduction or via hydrolysis to OxPt-bc followed by reduction (Fig. 3a, b). The dual release pathways for both DHA and OxPt may be beneficial in avoiding resistance mechanisms. Specifically, the OxPt-bc prodrug is resistant to deactivation by GSH and thiol-containing proteins, allowing for prolonged drug exposure when reduced to parent OxPt by ascorbate in cytosols and even nuclei of cancer cells.

Chemotherapies are often beleaguered by toxicities arising from accumulation in healthy tissues, which can be alleviated with nanoparticle delivery^38–40^. OxPt/DHA are optimally sized pegylated nanoparticles that are large enough to avoid renal filtration (~10 nm) but small enough to penetrate through the leaky tumour vasculatures in tumours^41–43^. The use of ~20 mol% PEG in this formulation helps to reduce plasma protein binding, thus minimizing MPS uptake after systemic injection^44–48^. OxPt/DHA showed low uptake in the liver and other major organs associated with clearance, and significantly increased both the single and repeated dose MTDs of OxPt. Mice were dosed near the free drug MTD, but only one tenth the single dose MTD of the NCP formulation, allowing for dose-dense metronomic chemotherapy. This significantly reduced the most common dose-limiting toxicity of peripheral neuropathy while maintaining strong anticancer efficacy and immunity, suggesting that OxPt/DHA may be a strong clinical candidate.

Nanoparticle-supported chemoimmunotherapy has been increasingly studied to alter the tumour microenvironment in recent years^49–52^. Though many chemotherapeutic agents mediate their cytotoxic effects by inducing immunologically silent or tolerogenic apoptosis, certain chemotherapies kill cancer cells via immunogenic apoptosis. This changes the cell-surface composition and releases DAMPs, of which there are three hallmarks: (i) the preapoptotic exposure of CRT on the cell surface, (ii) release of ATP during the blebbing phase of apoptosis, and (iii) postapoptotic exodus of the chromatin-binding protein HMGB-1 ^53^. While OxPt is a known ICD inducer, we have directly shown that treatment of CRC cells with DHA causes translocation of CRT to the cell surface and HMGB-1 release. This facilitates the recruitment of antigen-presenting cells (APCs) into the tumour bed (stimulated by ATP), the engulfment of dying tumour cells and their debris by APCs (stimulated by CRT)^23,54^, and optimal antigen presentation to T cells (stimulated by HMGB-1)^55,56^. DHA-treated CRC cells were uptaken more by phagocytes and led to strong T cell priming and antitumour vaccination. Altogether, these processes result in a potent IFN-γ-mediated immune response, which eventually can lead to tumour rejection^57^.

**Fig. 9:**
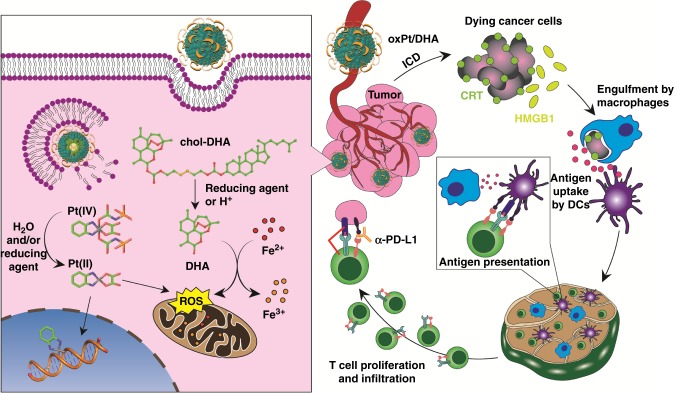
Anticancer and immune mechanisms of OxPt/DHA. left, The disruption of the lipid bilayer of OxPt/DHA upon endocytosis exposes the chol-DHA and OxPt prodrugs to triggered release by hydrolysis and/or reduction. The resultant parent drugs exhibit the expected mechanisms of action of DNA adduct formation and/or ROS generation. right, Systemically delivered OxPt/DHA can accumulate in the tumours and release the drug payload, as shown on the left, for immunogenic cell death of the cancer cells. Cell-surface CRT expression and release of DAMPs, such as HMGB1, lead to phagocytosis by macrophages and/or dendritic cells, which travel to the regional lymph node to prime T cells. Macrophages can release the antigens for DC uptake and subsequent T cell activation. The tumour-specific T cells proliferate and infiltrate into the tumour, where they can exert their cytotoxic effects. The α-PD-L1 prevents the binding of tumour PD-L1 and T cell PD-1, thereby inhibiting deactivation of the T cells

OxPt/DHA combined with α-PD-L1 is highly effective in treating well-developed tumours of CT26, likely due to innately high T cell and low suppressor cell infiltration^58,59^. This treatment led to eradication of 6/6 tumours and tumour-specific immune responses resulting in vaccination against subsequent live cell challenge. In contrast, the MC38 tumour model is highly immunosuppressive, with myeloid-derived suppressor cells constituting >50% of the CD45^+^ immune cells in tumours^58^. A higher dose of OxPt/DHA could approximately recapitulate the effects observed in CT26, with tumour eradication in 3/5 mice and long-term tumour control in the other two. Treatment with OxPt/DHA led to ICD in tumours followed by infiltration of and engulfment by phagocytes. These APCs can present tumour-specific antigens to naïve T cells in the lymph nodes and eventual T cell infiltration into the tumours as part of the adaptive immune response. Anti-PD-L1 ameliorates immune suppressive mechanisms of tumour cells causing immune evasion and T-cell anergy and/or exhaustion (Fig. 9)^60^.

In summary, we present an approach to initiate and stimulate immune-mediated eradication of cancer cells using synergistic nanomedicines. OxPt/DHA nanoparticles efficiently prevent the decomposition and degradation by encapsulation of and increase tumour uptake of DHA and OxPt prodrugs to harness the anticancer and immunostimulatory properties of ROS-producing DHA and OxPt. By inducing CRT exposure and HMGB-1 release, OxPt/DHA directly converted treated tumours into an in situ vaccine, recruiting antigen-presenting DCs and macrophages, facilitating cancer cell phagocytosis, enhancing antigen processing and presentation, and finally increasing intratumoural infiltration of CD8^+^ T cells to significantly potentiate checkpoint blockade immunotherapy. The animals treated by metronomic dosing of OxPt/DHA and α-PD-L1 were tumour-free for at least 3 months and immunized against live tumour cell challenge by generating long-term antitumour immunity. The scalable and tunable nature of NCP synthesis should allow their rapid optimization to lead to potential clinical candidates for combination therapy with immune checkpoint inhibitors.

## Methods

### Materials, cell lines, and animals

All starting materials were purchased from Sigma-Aldrich and Fisher (USA), unless otherwise noted, and used without further purification. 1,2-dioleoyl-snglycero-3-phosphate (DOPA), 1,2-dioleyl-sn-glycero-3-phosphocholine (DOPC), cholesterol, and 1,2-distearoyl-sn-glycero-3-phosphoethanolamine-N-[amino(polyethylene glycol)2000] (DSPE-PEG2k) were purchased from Avanti Polar Lipids (USA).

Murine colon adenocarcinoma cell CT26 and MC38 cells, mouse mammary carcinoma cell 4T1, mouse Lewis lung carcinoma cell LL/2 were all obtained from the American Type Culture Collection (ATCC, Rockville, MD). CT26 cells were cultured in RPMI 1640, MC38, 4T1 and LL/2 were grown in Dulbecco’s modified Eagle’s medium, respectively, supplemented with 10% FBS, 100 U/mL penicillin G sodium and 100 μg/mL streptomycin sulphate in a humidified atmosphere containing 5% CO_2_ at 37 °C. Mycoplasma was tested before use by MycoAlert detection kit (Lonza Nottingham, Ltd.).

BALB/c female mice (6 weeks, 18–22 g), C57BL/6 female mice (6 weeks, 18–22 g), Rag^2−/−^ female mice (6 weeks, 18–22 g), and SD/CD female rats (6 weeks, 160–200 g) were provided by Harlan-Envigo Laboratories, Inc (USA). We have complied with all ethical regulations for animal testing and research, with a study protocol reviewed and approved by the Institutional Animal Care and Use Committee (IACUC) at the University of Chicago.

### Preparation and characterization of OxPt/DHA

OxPt-bare was synthesized according to our previously reported method with minor modifications^61^. Briefly, an aqueous solution of OxPt-bp (5 mg, 25 mg/mL) was added to a 5 mL of 0.3 M Triton X-100/1.5 M 1-hexanol in cyclohexane and stirred vigorously for 15 min in the presence of DOPA (4 mg, 200 mg/mL in CHCl_3_). An aqueous solution of Zn(NO_3_)_2_ (20 mg, 100 mg/mL) was added to a 5 mL of 0.3 M Triton X-100/1.5 M 1-hexanol in cyclohexane and stirred vigorously for 5 min. The Zn(NO_3_)_2_-containing microemulsion was added dropwise to the Pt-containing microemulsion and stirred vigorously for 30 min at room temperature. After the addition of 10 mL ethanol, OxPt-bare was obtained by centrifugation at 11,628 × *g*. The resulting pellet was washed once with 50% cyclohexane/ethanol and once with THF/ethanol and finally redispersed in THF and filtered through a 200 nm syringe filter. The loadings of OxPt in the particles were determined by ICP-MS (Agilent 7700×, Agilent Technologies, USA) after digestion with nitric acid. OxPt/DHA was prepared by adding a THF solution (80 µL) of DOPC, cholesterol, DSPE-PEG2k, chol-DHA (3:1.5:1.5:1), and OxPt-bare to 500 µL of 30% (v/v) ethanol/water at 50 °C. OxPt/DHA particles with higher DHA:OxPt ratios were similarly prepared by increasing the amounts of chol-DHA. The mixture was stirred at 1700 rpm for 1 min. THF and ethanol were completely evaporated and the solution was allowed to cool down to room temperature. The particle size and zeta potential were determined by dynamic light scattering using a Zetasizer (Nano ZS, Malvern, UK). Transmission electron microscopy (TEM, Tecnai Spirit, FEI, USA) was used to observe the morphology. To determine chol-DHA loading, OxPt/DHA was centrifuged at 11,337 × *g* for 30 min, the supernatant was removed and the particles were resuspended in THF which dissolves the lipid layer to release chol-DHA. The amount of chol-DHA in the nanoparticle suspension was then determined by liquid chromatography-mass spectrometry (LC-MS, Agilent 6540, Agilent Technologies, USA). The stability of OxPt/DHA was evaluated at 4 °C in 5% dextrose or 37 °C in phosphate-buffered solution (PBS) containing BSA. The chol-DHA content in the nanoparticles was also determined by LC-MS before or after the addition of Triton X-100 to disrupt the lipid bilayer.

### In vitro cytotoxicity

CT26 cells or MC38 cells were seeded in 96-well plates at a density of 2 × 10^3^ cells per well and allowed to adhere for 24 h. Cells were then treated with different concentrations of free OxPt, free DHA, free OxPt plus DHA (OxPt + DHA), OxPt NCP, Zn/DHA, or OxPt/DHA at 1:0.5, 1:1 or 1:2 molar ratios of OxPt to DHA for another 72 h. Cell viability was detected by 3-(4,5-dimethylthiazol-2-yl)-5-(3-carboxymethoxyphenyl)-2-(4-sulfophenyl)-2H-tetrazolium assay (Promega, Madison, WI) according to the manufacturer’s instructions.

### Apoptosis analysis

CT26 cells seeded in six-well plates (5 × 10^4^ cells/well) were treated with free OxPt, free DHA, OxPt + DHA, OxPt NCP, Zn/DHA, or OxPt/DHA at a concentration of 10 μM OxPt or/and 5 μM DHA for 24 h, then harvested, washed twice with ice-cold PBS, stained with Alexa Fluor 488-Annexin V and propidium iodide (PI) for 15 min at room temperature in the dark, and then analysed by flow cytometry (LSR II, BD, USA).

### Cell cycle assay

Treated CT26 cells as described above were collected, washed twice with ice-cold PBS, fixed with 70% ethanol at 4 °C overnight and treated with RNase A for 45 min, and followed by PI staining for 30 min. The alteration of cell cycle was analysed by flow cytometry.

### ROS generation

CT26 cells were treated with free OxPt, free DHA, OxPt + DHA, OxPt NCP, Zn/DHA, or OxPt/DHA at a concentration of 10 μM OxPt or/and 5 μM DHA for 24 h, then incubated with 10 μM H_2_DCFDA (Thermo Fisher, USA) for another 1 h. The cells were collected, washed twice with ice-cold PBS, and analysed by flow cytometry.

### Cytochrome *c* release

After treatment with free OxPt, free DHA, OxPt + DHA, OxPt NCP, Zn/DHA, or OxPt/DHA at a concentration of 10 μM OxPt or/and 5 μM DHA for 24 h, CT26 cells were stained with MitoTracker Red CMXRos (100 μM) for 1 h, then fixed with 4% paraformaldehyde for 10 min, permeabilized with 0.2% Triton X-100 for 10 min, incubated with anti-cytochrome *c* (eBioscience, diluted 1:100) for 2 h, stained with DAPI for 10 min, and observed under CLSM (Olympus, FV1000).

### CRT exposure analysis

CT26 cells seeded in six-well plates (2 × 10^5^ cells/well) were cultured with free drugs or nanoparticles at a dose of 10 μM OxPt or/and 5 μM DHA for 24 h. The treated cells were collected, incubated with Alexa Fluor 488-CRT antibody (Enzo cat # ADI-SPA-601-488-F, diluted 1:100) for 2 h, stained with PI, and analysed by flow cytometer to identify CRT exposure. The fluorescence intensity of stained cells was gated on PI^−^ cells.

For surface detection of CRT, CT26 cells were seeded on 10 mm^2^ glass coverslips placed in six-well plates at a density of 2 × 10^5^ cells per well. After treatment, cells were washed with PBS three times, incubated with Alexa Fluor 488-CRT antibody (diluted 1:100) for 2 h, stained with DAPI, and observed under CLSM using 405 nm and 488 nm lasers for visualizing nuclei and CRT expression on the cell membrane, respectively.

### Detection of HMGB-1 release

CT26 cells seeded in six-well plates (2 × 10^5^ cells/well) were cultured with free drugs or nanoparticles at a dose of 10 μM OxPt or/and 5 μM DHA for 24 h. The medium was collected for detection of HMGB-1 release by ELISA according to the manufacturer’s instructions (Chondrex, Redmond, WA).

### Phagocytosis assay

Bone-marrow-derived dendritic cells and macrophages were isolated according to previously published protocols^62,63^. Briefly, the hind legs of mice were cut away just above the hip and below the ankle. The muscle was cut away and the bones were soaked in 70% ethanol for 30 s. In a sterile environment, the ends of each bone were cut away and an insulin syringe filled with RPMI complete media was inserted into the exposed end of the bone and used to flush out the bone marrow into a cell culture dish filled with RPMI complete media. This process was repeated until the bone appeared white and translucent. For DC activation, bone-marrow-derived monocytic cells were cultured with GM-CSF (20 ng/mL) and IL-4 (10 ng/mL) for 6 days, then nonadherent cells in the culture supernatant were harvested and the expression of CD11c, CD11b, F4/80 and Gr-1 was analysed by flow cytometry to determine the purity of DC before further use. For macrophage differentiation, bone-marrow-derived monocytic cells were cultured with M-CSF (20 ng/mL) for 6 days, then adherent cells were harvested and the expression of CD11c, CD11b and F4/80 was analysed by flow cytometry to determine the purity of macrophage before further use. TdTamato-transfected MC38 tumour cells were first incubated with OxPt/DHA at 10 μM OxPt and 5 μM DHA for 24 h, then treated tumour cells were cocultured with F4/80-labelled (eBioscience) macrophages or CD11c-labelled (eBioscience) DCs at ratio of 1:1 for 4 h at 37 °C. Cells were then collected, washed twice with cold PBS, resuspended in PBS, analysed by flow cytometry, and calculated as the percentage of TdTamato^+^ cells within F4/80^+^ macrophages or CD11c^+^ DCs.

### Antigen presentation

Bone-marrow-derived dendritic cells or macrophages were cocultured with PBS-, OxPt-, DHA-, OxPt + DHA-, or OxPt/DHA-treated MC38 cells. After 48 h, the cells were washed twice with PBS and stained with anti-CD16/32 (clone 93; eBiosciences) to reduce nonspecific binding to FcRs followed by CD11b (M1/70), CD11c (N418), F4/80 (BM8), and SIINFEKL/H-2Kb (25-D1.16), and yellow fluorescence dye (all from eBioscience).

### Priming assay

1 × 10^6^ MC38 cells were treated with 100 μM OxPt or/and 50 μM DHA for 24 h, and then injected into the footpads of C57Bl/6 mice. Six days later, popliteal lymph node cells were collected by homogenizing and filtering the organ through a sterile cell strainer (40 μm; Fisher Scientific). 1 × 10^5^ lymph node cells were cultured in complete culture medium in the presence of MC38 cell lysates killed by freeze-thaw cycle in 200 μL medium in round-bottom 96-well plates. Three days later, the supernatants were harvested and IFN-γ secretion was determined by ELISA (eBioscience). The ability of OxPt/DHA-treated MC38 cell lysates to enhance the secretion of INF-γ by T cells was also compared with a known antigen KSPWFTTL and the positive control CD3ɛ plus CD28.

### Antitumour vaccination

1 × 10^6^ MC38 cells treated with 100 μM OxPt or/and 50 μM DHA for 24 h were subcutaneously inoculated into the lower flank of 6-week-old female C57Bl/6 mice or Rag^2−/−^ mice. Seven days later, 2 × 10^5^ living MC38 cells were inoculated into the contralateral flank. Mice were then monitored for the appearance of tumours for 30 days.

### In vivo pharmacokinetics and biodistribution analysis

SD/CD rats were intravenously (i.v.) injected with OxPt/DHA at an OxPt dose of 6 mg/kg (2.14 mg/kg DHA). The blood was collected at 5 min, 30 min, 1 h, 3 h, 5 h, 8 h, 24 h, and 48 h post injection and immediately centrifuged at 604 × g for 10 min to harvest plasma samples. Twenty-five microliters plasma was digested with concentrated nitric acid for 24 h and analysed for Pt concentration by ICP-MS. Another 25 μL plasma was added to 5 μL 20% Triton X-100 to disrupt the lipid bilayer of the nanoparticles, chol-DHA was then extracted from plasma by adding 100 μL ethyl acetate, followed by centrifugation at 6708 × *g* for 10 min. The chol-DHA content was quantified by LC-MS.

BALB/c mice were subcutaneously injected in the right flank with 1 × 10^6^ CT26 cells. When the tumours reached ~100 mm^3^, mice were intraperitoneally (i.p.) administrated OxPt/DHA at an OxPt dose of 8 mg/kg (2.86 mg/kg DHA). The blood was collected at 1, 3, 5, 8, 24, 48, and 72 h post injection and immediately centrifuged at 604 × *g* for 10 min to harvest plasma samples. The content of Pt and chol-DHA was quantified by ICP-MS and LC-MS, respectively. The livers, lungs, spleens, kidneys, bladders, and tumours were also harvested, digested with concentrated nitric acid for 24 h, and analysed for Pt concentration by ICP-MS.

### In vivo toxicity on mice

Balb/c mice received i.p. doses of Zn/DHA at 5 mg/kg DHA every 3 days for a total of 50 doses. C57Bl/6 mice received a weekly i.p. dose of OxPt/DHA at 60 mg/kg OxPt (21.5 mg/kg DHA) for a total of four doses or a single i.p. dose at 80 mg/kg OxPt (28.6 mg/kg DHA). The activity level and body weights of the mice were monitored for toxicity.

SD/CD rats received weekly i.v. doses of OxPt/DHA or free OxPt at 8 mg/kg OxPt (2.86 mg/kg DHA) for a total of three doses to measure peripheral neuropathy. A hind paw was subjected to a constant heat source through a 3/8″ glass pane and tested for the time to withdrawal to measure peripheral neuropathy.

### In vivo anticancer efficacy

1 × 10^6^ cells CT26 or MC38 cells were subcutaneously injected into the right flank region of 6-week BALB/c, C57Bl/6 wild-type or Rag^2−/−^ mice, respectively. Twelve days after tumour inoculation, mice were i.p. dosed with 8 mg/kg OxPt, 2.86 mg/kg DHA, and/or 75 μg PD-L1 antibody once every 3 days for up to 12 doses. Tumour growth was monitored by measurement with a digital caliper, where tumour volumes were calculated as follows: (width^2^ × length)/2.

Tumour-free BALB/c mice were challenged with 5 × 10^6^ CT26 cells on the contralateral flank 3 months after the tumours disappeared. The mice were monitored for 1 month and then rechallenged with 5 × 10^4^ unrelated 4T1 cells.

### Immunofluorescence assay

Tumours were collected 2 days or 12 days after the first treatment, and frozen tissue sections of 5 µm thickness were prepared using a cryostat. The sections were fixed in acetone for 10 min at −20 °C, blocked with 2% BSA for 1 h, and incubated with individual primary antibodies against CD11c (eBioscience), F4/80 (eBioscience), CD3ɛ (Santa Cruz) or CD8 (Thermo Scientific) overnight at 4 °C, followed by incubation with dye-conjugated secondary antibodies for 1 h at room temperature. After staining with DAPI for another 10 min, the sections were then washed twice with PBS and observed under CLSM.

### Flow cytometry assay for immune response

Tumours were harvested on 12 days after the first treatment, treated with 1 mg/mL collagenase I (Gibco™, USA) for 1 h, and ground with the rubber end of a syringe. Cells were filtered through nylon mesh filters and washed with PBS. The single-cell suspension was incubated with anti-CD16/32 (clone 93; eBiosciences) to reduce nonspecific binding to FcRs. Cells were further stained with the following fluorochrome-conjugated antibodies: CD45 (30-F11), CD3ɛ (145–2C11), CD8 (53–6.7), CD11b (M1/70), CD11c (N418), F4/80 (BM8), MHC II (AF6–120.1), CD86 (PO3), CD206 (C068C2), CD44 (IM7), CD62L (MEL-14), and Zombie NIR (all from eBioscience). LSR FORTESSA (BD Biosciences) was used for cell acquisition, and data analysis was carried out using FlowJo software (TreeStar, Ashland, OR). All antibodies were diluted 1:200 for use.

### ELISPOT assay

Tumour-specific immune responses to IFN-γ was measured in vitro by an ELISPOT assay (Mouse IFN-γ ELISPOT Ready-SET-Go!®; Cat. No. 88-7384-88; eBioscience). A Millipore Multiscreen HTS-IP plate was coated overnight at 4 °C with anti-Mouse IFN-γ capture antibody (diluted 1:250). Single-cell suspensions of splenocytes were obtained from MC38 tumour-carrying mice on 12 days after the first treatment and seeded onto the antibody-coated plate at a concentration of 2 × 10^5^ cells/well. Cells were incubated with or without KSPWFTTL stimulation (10 µg/mL; in purity 495%; PEPTIDE 2.0) for 48 h at 37 °C and then discarded. The plate was then incubated with biotin-conjugated anti-IFN-γ detection antibody (diluted 1:250) at room temperature for 2 h, followed by incubation with Avidin-HRP for 2 h at room temperature. 3-amino-9-ethylcarbazole (AEC) substrate solution (Sigma, Cat. AEC101) was added for cytokine spot detection.

### Reporting summary

Further information on experimental design is available in the Nature Research Reporting Summary linked to this article.

## Supplementary information

Supplementary Information

Reporting Summary

## Data Availability

The authors declare that all the data supporting the findings of this study are available within the article and its Supplementary Information files or from the corresponding author upon reasonable request. The crystal structure reported is deposited at the Cambridge Crystallographic Data Centre (CCDC) under deposition number CCDC 1875999. The crystallographic file can be obtained free of charge from the Cambridge Crystallographic Data Centre via http://www.ccdc.cam.ca.uk/data_request/cif.
