# Supplementary material for: Immunostimulatory nanomedicines synergize with checkpoint blockade immunotherapy to eradicate colorectal tumors
Source: Nat Commun. 2019 Apr 23;10:1899. doi: 10.1038/s41467-019-09221-x (PMC6478897; doi:10.1038/s41467-019-09221-x)
Supplement: Supplementary file 1 — Supplementary Information [file 41467_2019_9221_MOESM1_ESM.pdf]

Immunostimulatory Nanomedicines Synergize with Checkpoint Blockade Immunotherapy to Eradicate Colorectal Tumors

Xiaopin Duan,[1,†] Christina Chan,[1,†] Wenbo Han,[1,†] Nining Guo,[1,2] Ralph R. Weichselbaum,[2] Wenbin Lin[1,2*]

[1]Department of Chemistry, The University of Chicago, 929 E 57th St, Chicago, IL 60637, USA.

[2]Department of Radiation and Cellular Oncology and Ludwig Center for Metastasis Research, The University of Chicago 5758, S Maryland Ave, Chicago, IL 60637, USA.

[*]Corresponding author. E-mail: wenbinlin@uchicago.edu

[†]These authors contributed equally to this work.

Supplementary Method 1: Synthesis of OxPt prodrug.

The synthesis of OxPt prodrug is shown in Supplementary Figure 1. 3.0 g oxaliplatin (OxPt) and 30mL deionized water were charged to a 50 mL round bottom flask with a stir bar. The mixture was stirred and cooled over an ice bath to 0ºC. 5.45 mL 30% hydrogen peroxide was slowly added to the mixture in 15 min. The reaction mixture was stirred in the dark overnight at room temperature. The mixture was carefully concentrated to approximately 5 mL on a rotary evaporator. 25 mL ethanol was added and the mixture was cooled at -20ºC for 3 h. The white solid was collected by vacuum filtration followed by washing with cold water (2×20 mL) and cold ethanol (2×20 mL) to give a quantitative yield (3.25 g) of the OxPt-$(OH)_2$ intermediate. ^{1}H-NMR (500 MHz, D_2O): 1.16 (t, 2H), 1.47 (d, 2H), 1.53 (d, 2H), 2.17 (d, 2H), 2.74 (d, 2H).

3.0 g OxPt-$(OH)_2$ was charged to an oven-dried 50 mL round bottom flask with a stir bar. 10 mL anhydrous DMF was added to the flask and the flask was purged with N_2 for 30 min. The mixture was cooled over an ice bath for 15 min and then diethoxyphosphinyl isocyanate (2.2 mL, 2.55 g, 2.05 eq.) was added dropwise through a syringe. The mixture was then slowly warmed to room temperature and stirred in the dark overnight. The resulting mixture was directly loaded on a silica column and purified using 1:1 v/v DCM/hexanes and 10% methanol in DCM as the eluents to afford OxPt-ester. Yield: 4.56 g (83%). ^{1}H-NMR (500 MHz, DMSO-d_6): 1.12 (t, 2H), 1.26 (t, 12H), 1.50 (d, 2H), 1.60 (d, 2H), 2.15 (d, 2H), 2.73 (m, 2H), 4.00 (m, 8H), 7.96 (s, 2H), 8.41 (d, 2H), 8.75 (d, 2H).

1.0 g OxPt-ester was charged to an oven-dried 50 mL round bottom flask with a stir bar. 20 mL anhydrous DCM was added and the flask was purged with N_2 for 30 min. The flask was cooled over an ice bath for 15 min. 2.5 mL bromotrimethylsilane in 5 mL DCM was added dropwise through a syringe. The reaction mixture was slowly warmed to room temperature and stirred in the dark overnight. The solvent was then removed by rotary evaporation and further dried under high vacuum for 2 h. Then 5 mL cold methanol was added to the flask and stirred for 30 min at room temperature. The OxPt-bp product was crushed out by adding 30 mL DCM to the methanol solution and collected by centrifugation at 13000 rpm for 10 min. Yield: 0.59 g (69%). ^{1}H-NMR (500 MHz, D_2O): 1.19 (t, 2H), 1.51 (s, 4H), 2.19 (d, 2H), 2.90 (d, 2H).

Supplementary Method 2: Synthesis of chol-DHA.

The synthesis of chol-DHA prodrug is shown in Supplementary Figure 2. To a mixture of Cholesterol (500 mg, 1.3 mmol) and 4-N,N-dimethylaminopyridine (DMAP, 0.32 g, 2.8 mmol) in anhydrous DCM, a solution of triphosgene (128 mg, 0.43 mmol) in anhydrous DCM (2 mL) was added dropwise over an ice bath with stirring. The resultant solution was warmed to room temperature and further stirred for 20 min and then added dropwise to a solution of bis(2-hydroxyethyl) disulfide (440 mg, 2.8 mmol) in anhydrous DCM (5 mL) over an ice bath. The reaction mixture was then warmed to room temperature and stirred for 12 h. After removal of solvent, the residue was purified to afford cholest-5-en-3-ol (3β)-, 3-(2-((2-hydroxyethyl)disulfanyl)ethyl) carbonate (Chol-S-S-OH) by column chromatography on silica gel with ethyl acetate/hexane (1:2, v/v). Yield: 0.44 g (60%). ^{1}H-NMR (500 MHz, $CDCl_3$): 0.65 (s, 3H), 0.83 (d, 3H), 0.84-1.15 (m, 13H), 1.22-1.66 (m, 13H), 1.75-2.02 (m, 5H), 2.37 (m, 2H), 2.85 (m, 3H), 2.92 (t, 2H), 3.83 (t, 2H), 4.34 (t, 2H), 4.44 (m, 1H), 5.37 (d, 1H).

To a mixture of Chol-S-S-OH (83.5 mg, 0.15 mmol) and DMAP (54 mg, 0.44 mmol) in anhydrous DCM (2 mL), a solution of triphosgene (15 mg, 0.05 mmol) in anhydrous DCM (1 mL) was added dropwise over an ice bath with stirring. The resultant solution was warmed to room temperature and further stirred for 1 h and then added dropwise to a solution of DHA (50 mg, 0.17 mmol) in anhydrous DCM (5 mL) over an ice bath. The reaction mixture was then warmed to room temperature and stirred for 12 h. After removal of solvent, the residue was purified by column chromatography on silica gel with hexane/ethyl acetate (7:1, v/v) to yield 53 mg of Chol-DHA. ^{1}H-NMR (500 MHz, $CDCl_3$): 0.65 (s, 3H), 0.88-1.20 (m, 29H), 1.30-1.75 (m, 20H), 1.80-2.08 (m, 8H), 2.37 (m, 3H), 2.61 (m, 1H), 3.00 (m, 4H), 4.41 (m, 4H), 4.52 (m, 1H), 5.42 (d, 1H), 5.47 (s, 1H), 5.60 (d, 1H). ESI-MS: m/z=899.5 (expected 899.5 for $[M+Na]^+$).

Supplementary Method 3: Synthesis of chol-pyro.

The synthesis of chol-pyro prodrug is shown in Supplementary Figure 7. Pyropheophytin a was prepared as previously reported.[1] A mixture of pyropheophytin a (100 mg, 0.187 mmol), Chol-SS-OH (210 mg, 0.37 mmol), EDCI (72 mg, 0.37 mmol), DMAP (10 mg, 0.08 mmol) and DIPEA (100 μL) was stirred in anhydrous DCM (5 mL) at room temperature for 24 h. The mixture was loaded directly on a DCM-packed silica gel column and purified with a gradient elution from 5:1 DCM: EtOAc to 3:1 DCM: EtOAc to afford pure chol-pyro. Yield: 162 mg (79.8%). ^{1}H-NMR (500 MHz, $CDCl_3$): -1.33 (s, 2H), 0.59 (s, 3H), 0.81-1.99 (m, 18H), 1.20-1.42 (m, 16H), 1.50-1.95 (m, 12H), 2.25-2.47 (m, 4H), 2.62 (m, 1H), 2.81 (t, 2H), 2.90 (t, 2H), 3.24 (s, 3H), 3.43 (s, 3H), 3.68 (m, 4H), 4.33 (m, 6H), 4.52 (q, 2H), 5.12 (d, 1H), 5.16 (s, 1H), 5.30 (d, 1H), 6.18 (d, 1H), 6.32 (d, 1H), 8.01 (dd, 1H), 8.58 (s, 1H), 9.38 (s, 1H), 9.48 (s, 1H). ESI-MS: m/z=1083.6 (expected 1083.6 for $[M+H]^+$).

Supplementary Method 4: Preparation of fluorescently labeled NCP particle.

0.5 g OxPt-bp was mixed with 1.52 mL water and 0.98 mL 3 M NaOH to prepare a neutralized OxPt-bp stock solution. The solution was added to excess amount of xylenol orange solid to prepare a saturated solution. The saturated solution was centrifuged at 12000 rpm for 5 min to remove any solid before used to prepare bare NCP particle.

Bare particle was prepared according to our previously reported method by changing the prodrug solution to xylenol orange-saturated prodrug solution. Coated fluorescent particle was prepared by

the same method as OxPt/DHA particle. 30% of DOPC was changed to DOPE-FITC and all Chol-DHA was changed to chol-pyro.

Supplementary Method 5: Single crystal analysis of OxPt-bc.
Single crystal X-ray diffraction of was performed with a Bruker D8 Venture, dual microsource (Cu and Mo) diffractometer with a CMOS detector. Mo Kα radiation was used. Data reduction and integration were performed with the Bruker APEX3 software package (Bruker AXS, version 2015.5-2, 2015). Data were scaled and corrected for absorption effects using the multi-scan procedure as implemented in SADABS (Bruker AXS, version 2014/5, 2015, part of Bruker APEX3 software package). The structure was solved by SHELXT (Version 2014/5)[2] and refined by a full-matrix least-squares procedure using OLEX2[3] software packages (XL refinement program version 2014/7).[4] All non-hydrogen atoms are refined anisotropically. Crystallographic data and details of the data collection and structure refinement are listed in Supplementary Table 3.

Supplementary Method 6: Surgery control. 1×10^6 cells CT26 were subcutaneously injected into the right flank region of 6-week BALB/c. 12 days after tumour inoculation, mice were anesthetized for surgical removal of the tumours with skin glue to close the wound. Two weeks after surgery, 5×10^6 live CT26 cells were subcutaneously injected into the left flank and monitored for tumour development and growth, as previously described.

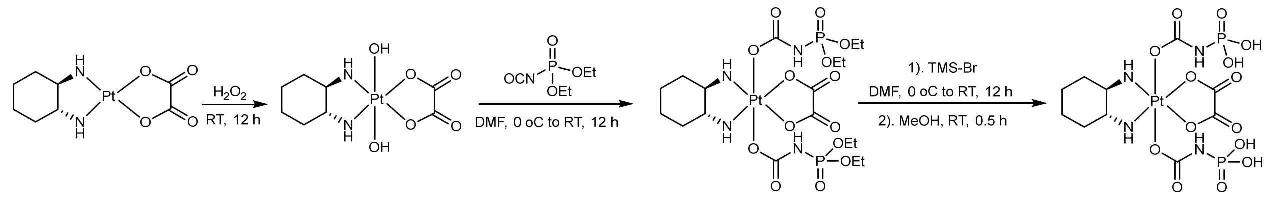

Supplementary Figure 1. Synthesis of OxPt-bp.

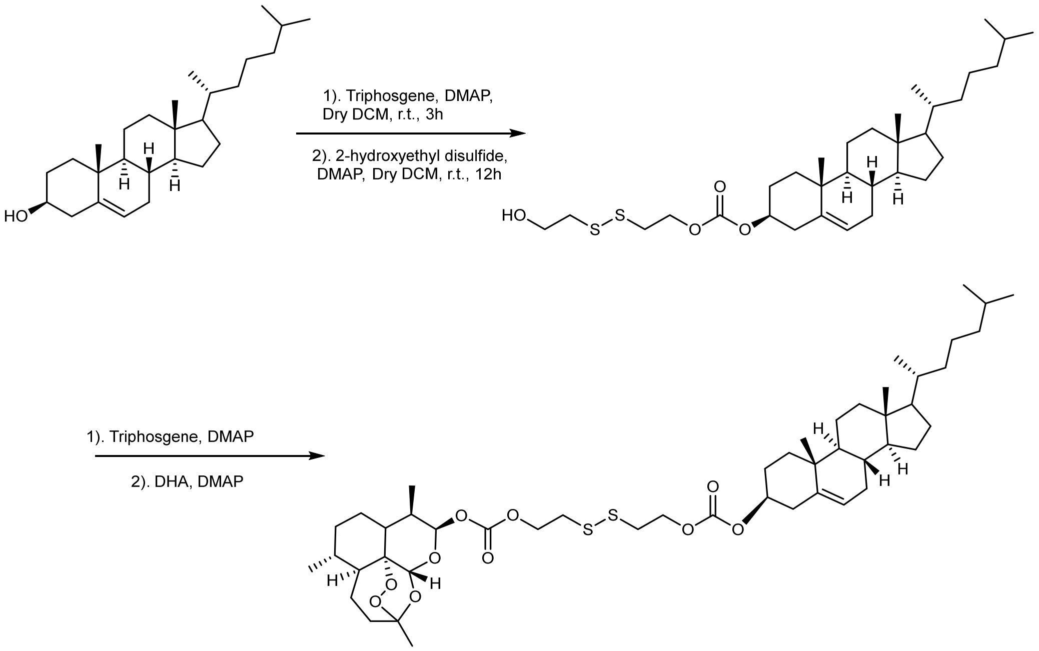

Supplementary Figure 2. Synthesis of chol-DHA.

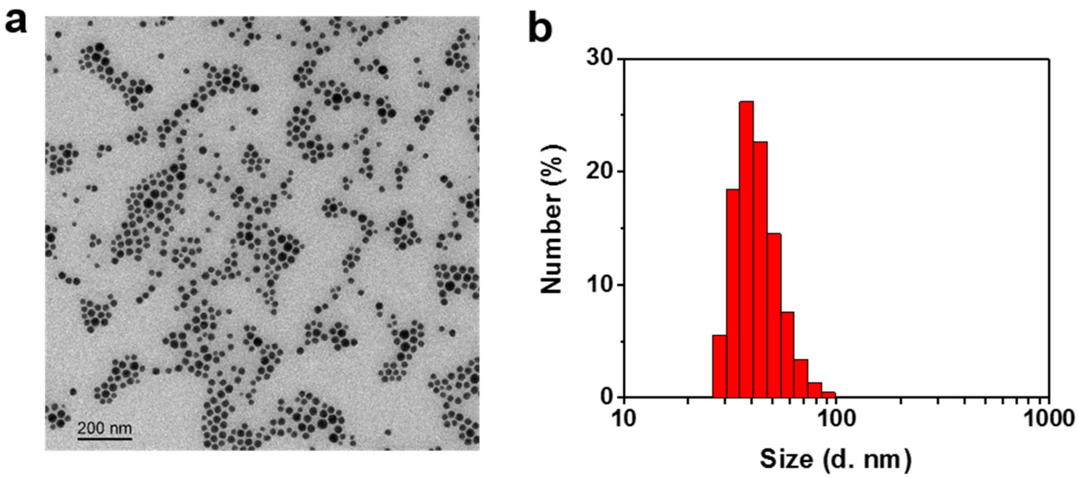

Supplementary Figure 3. a, TEM image of OxPt-bare. b, Number-average diameter of OxPt-bare characterized by DLS.

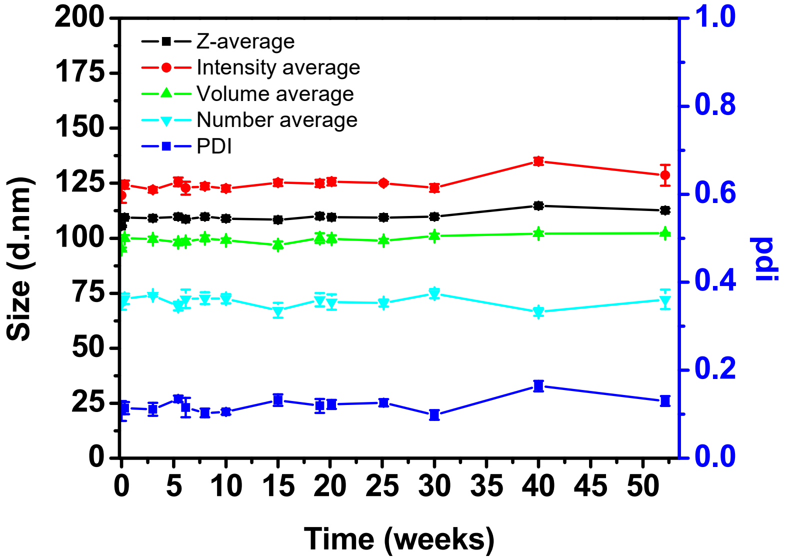

Supplementary Figure 4. Stability analysis of OxPt/DHA at 4 ºC.

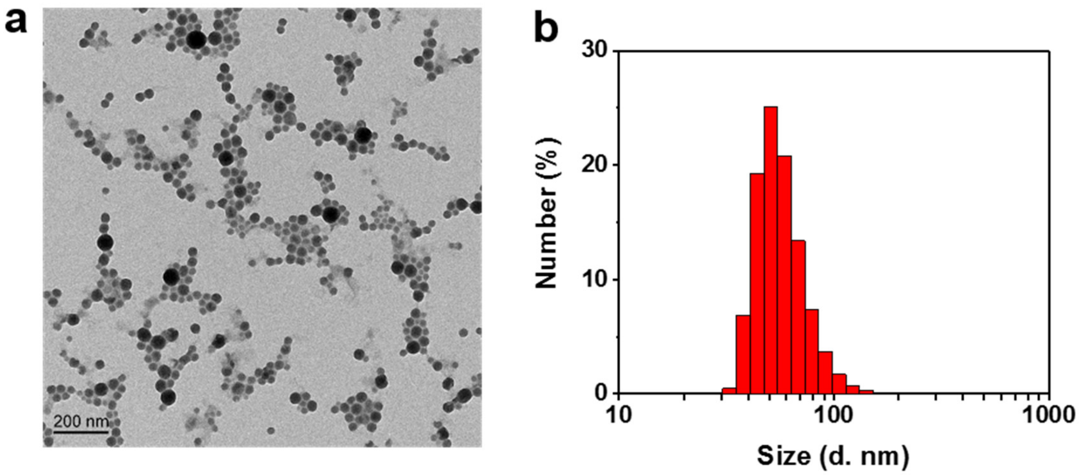

Supplementary Figure 5. a, TEM image of OxPt NCP. b, Number-average diameter of OxPt NCP characterized by DLS.

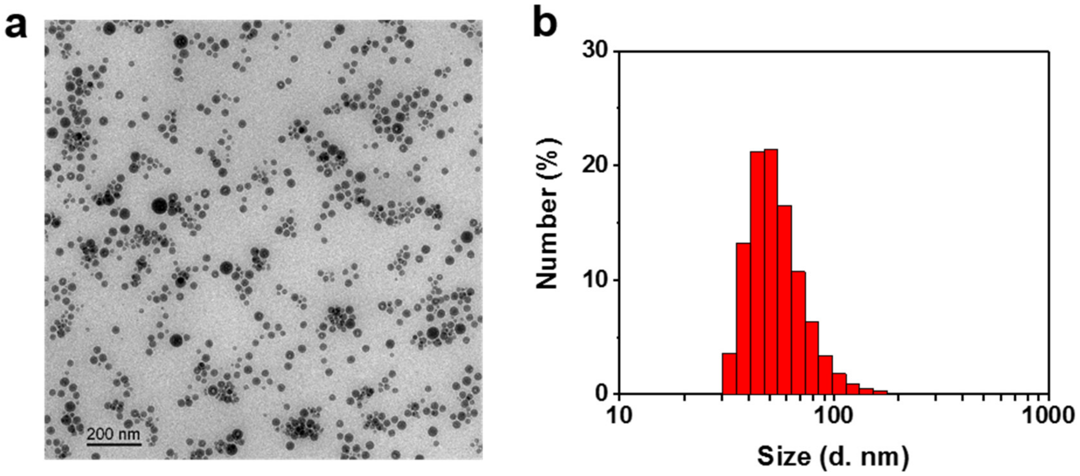

Supplementary Figure 6. a, TEM image of Zn/DHA. b, Number-average diameter of Zn/DHA characterized by DLS.

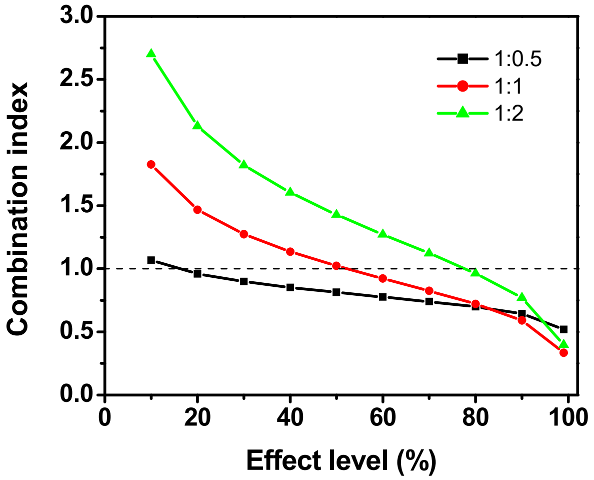

Supplementary Figure 7. Combination Index of OxPt and DHA NCP at different effect levels.

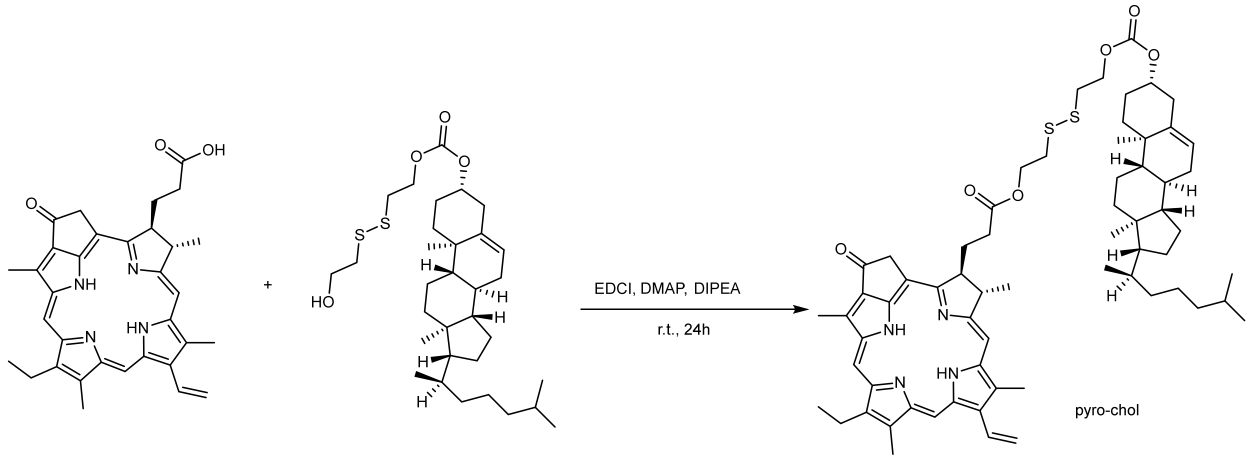

Supplementary Figure 8. Synthesis of chol-pyro.

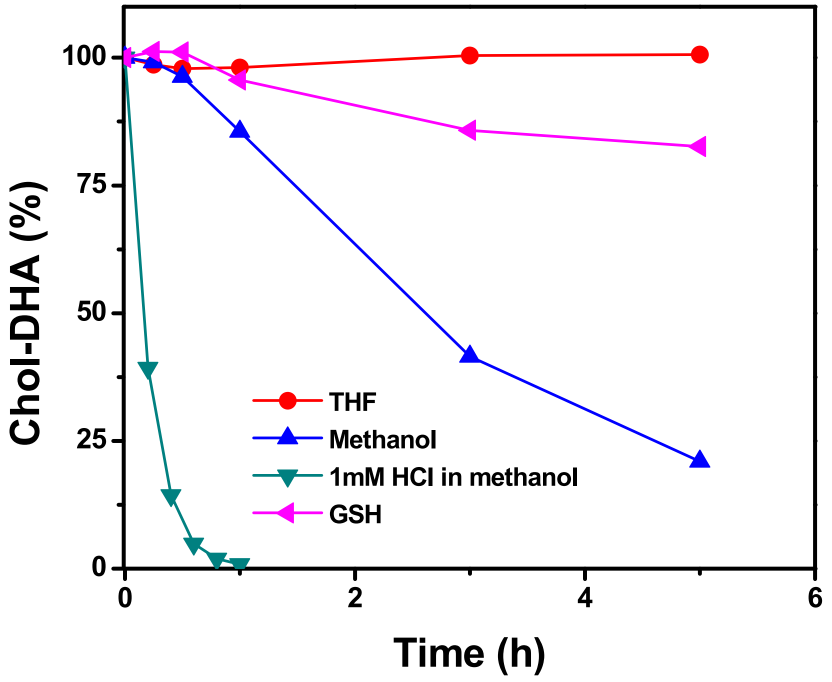

Supplementary Figure 9. Chol-DHA remaining in different solvents when incubated at 37°C. GSH curve was calculated by subtraction of the curve in water from the curve for 5 mM GSH water solution to show the DHA release by GSH reduction only.

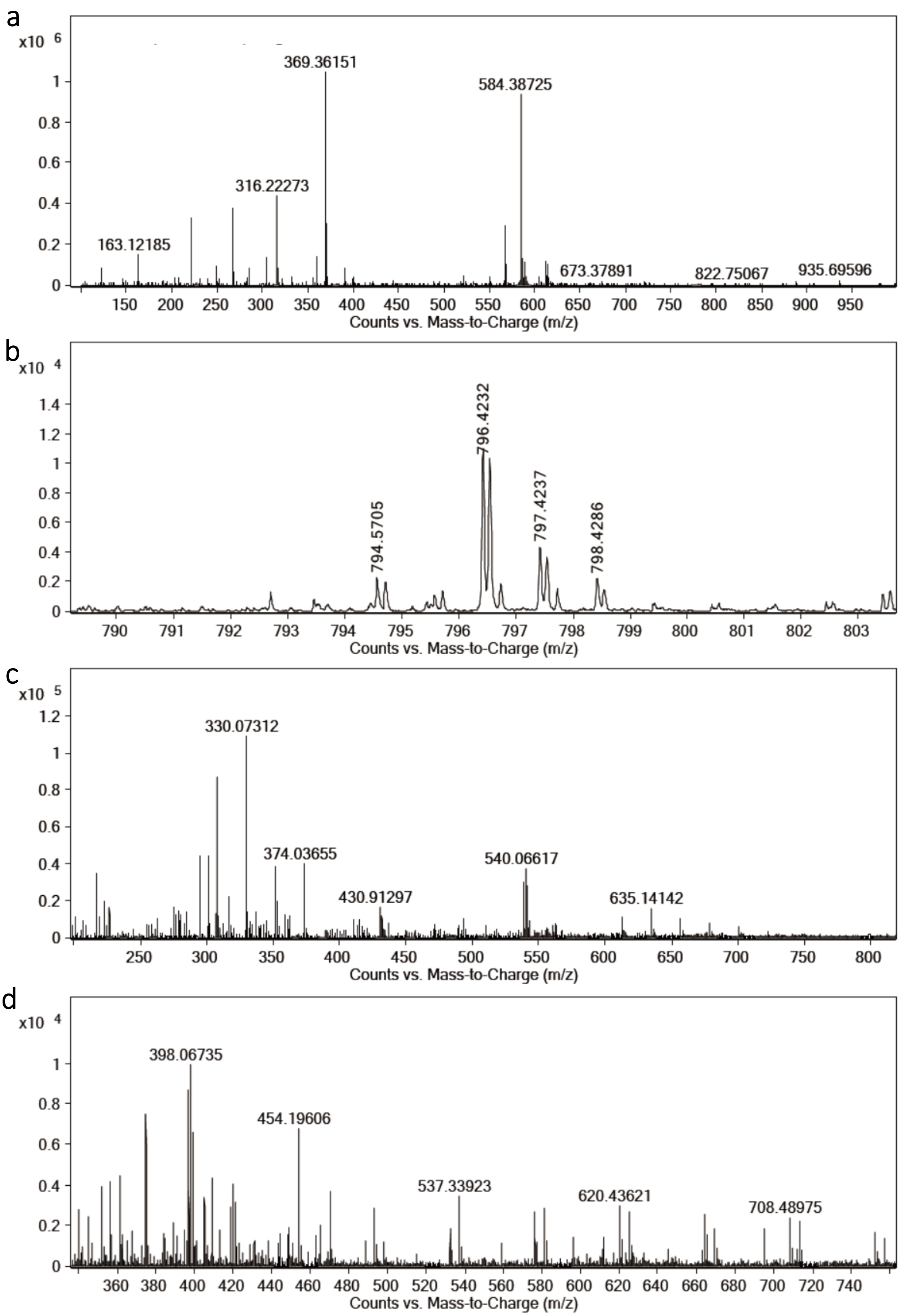
a
x10 6
369.36151
584.38725
316.22273
163.12185
673.37891
822.75067
935.69596
Counts vs. Mass-to-Charge (m/z)
b
x10 4
796.4232
797.4237
794.5705
798.4286
Counts vs. Mass-to-Charge (m/z)
c
x10 5
330.07312
374.03655
540.06617
430.91297
635.14142
Counts vs. Mass-to-Charge (m/z)
d
x10 4
398.06735
454.19606
537.33923
620.43621
708.48975
Counts vs. Mass-to-Charge (m/z)

Supplementary Figure 10. HR-MS showing the release mechanisms of drug release. a, chol-DHA decomposition product in methanol. 584.38725: Chol-S-S-OH; 369.36151: Chol-S-S-OH fragment; 316.22723: artemether. b, chol-DHA disulfide exchange product with GSH. 796.4232: Chol-S-GSH. c, hydrolysis product released from particle core (540.06617). d, reduction product (free oxaliplatin) of particle core by ascorbate (398.06735).

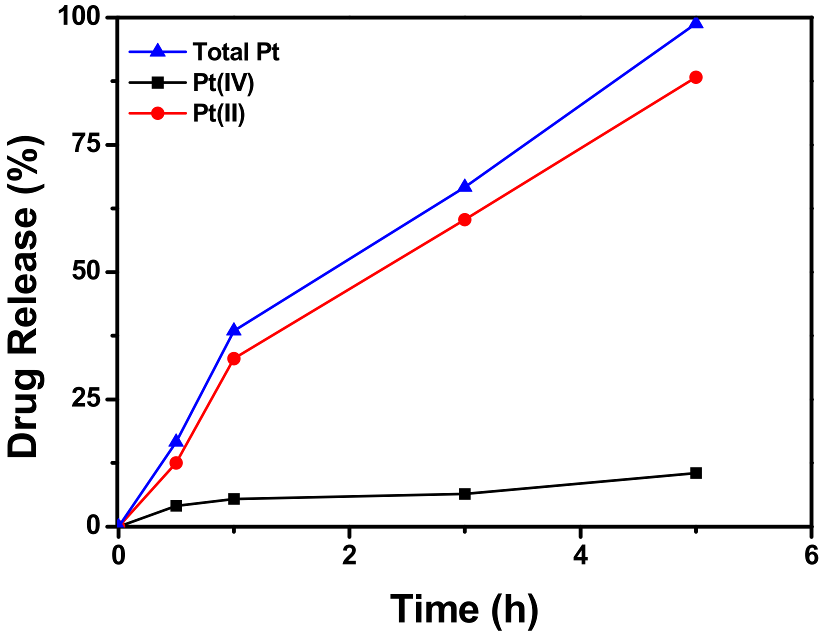

Supplementary Figure 11. Pt compounds released from OxPt/DHA particles when incubated in water at 37°C with 0.5% Triton X-100 and 5 mM ascorbate.

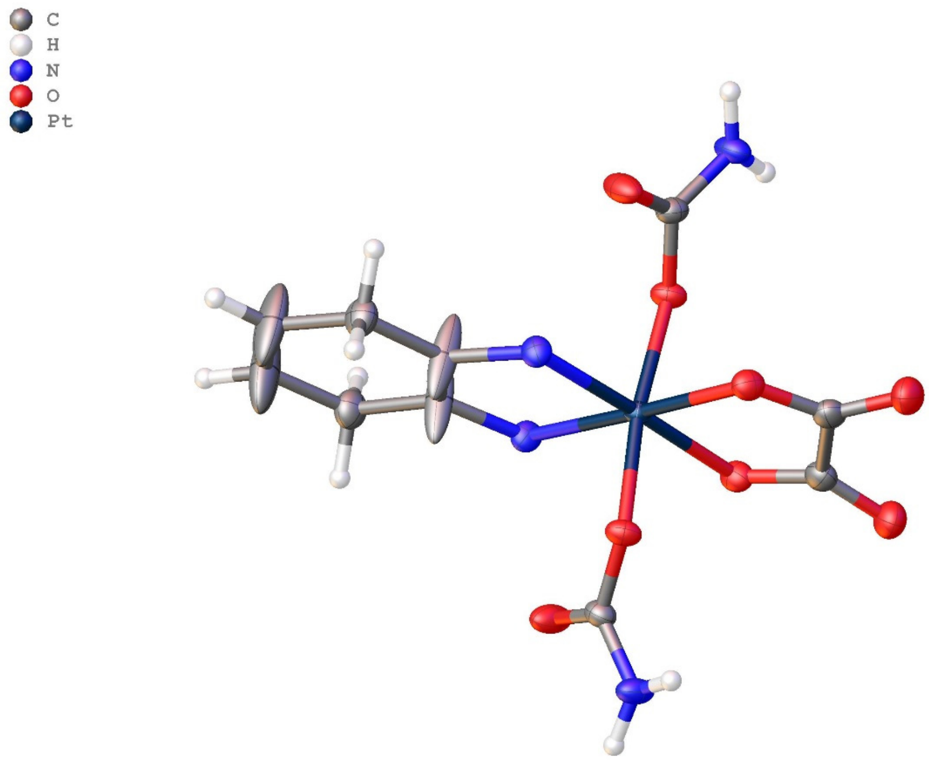

Supplementary Figure 12. Crystal structure of OxPt-bc (Pt: navy blue, O: red, N: blue, C: grey, H: white).

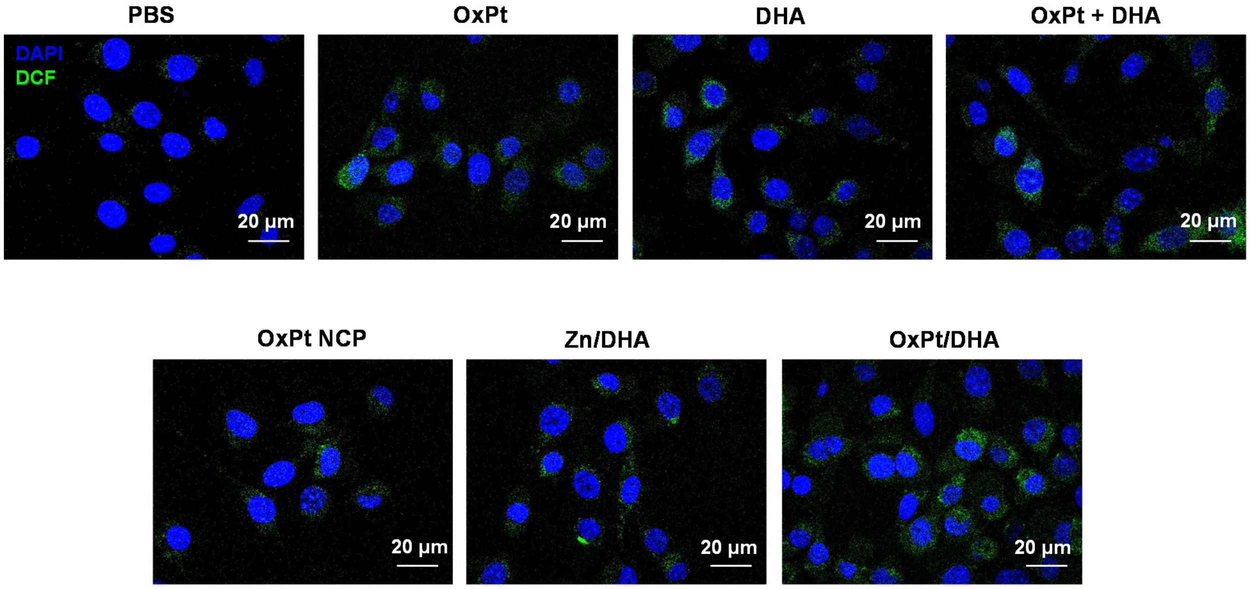

Supplementary Figure 13. ROS generation in CT26 cells incubated with OxPt, DHA or combinations.

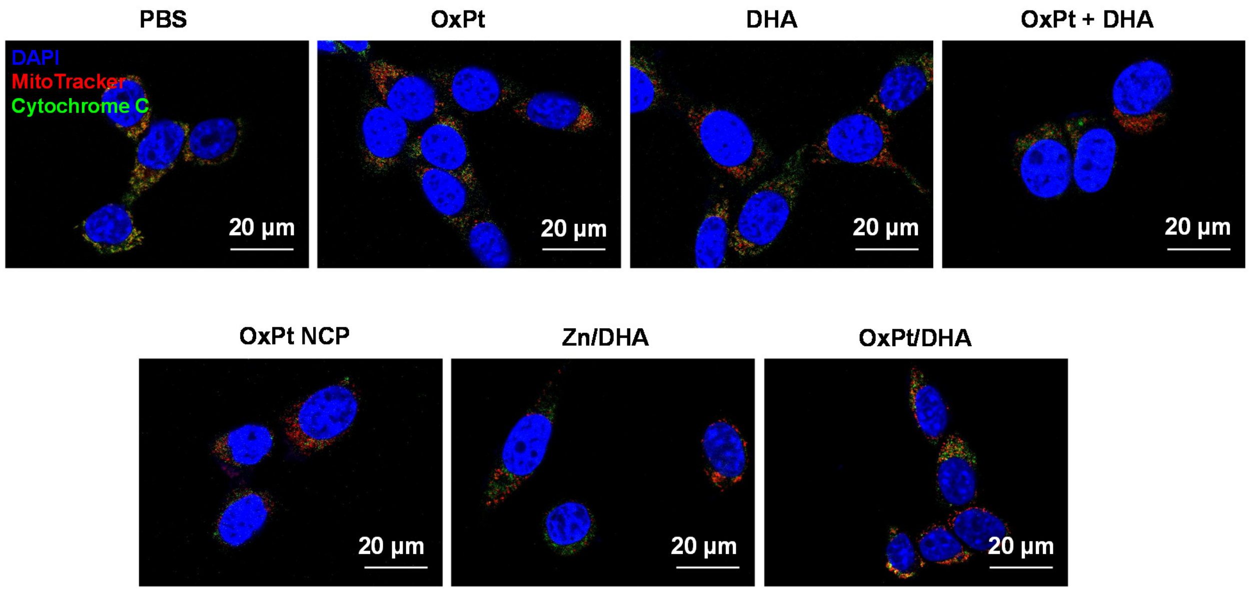

Supplementary Figure 14. Confocal images showing the cytochrome C (Green) release from mitochondria (Red) induced by OxPt, DHA or combinations, as evidenced by the decreased colocalization of green fluorescence with red fluorescence.

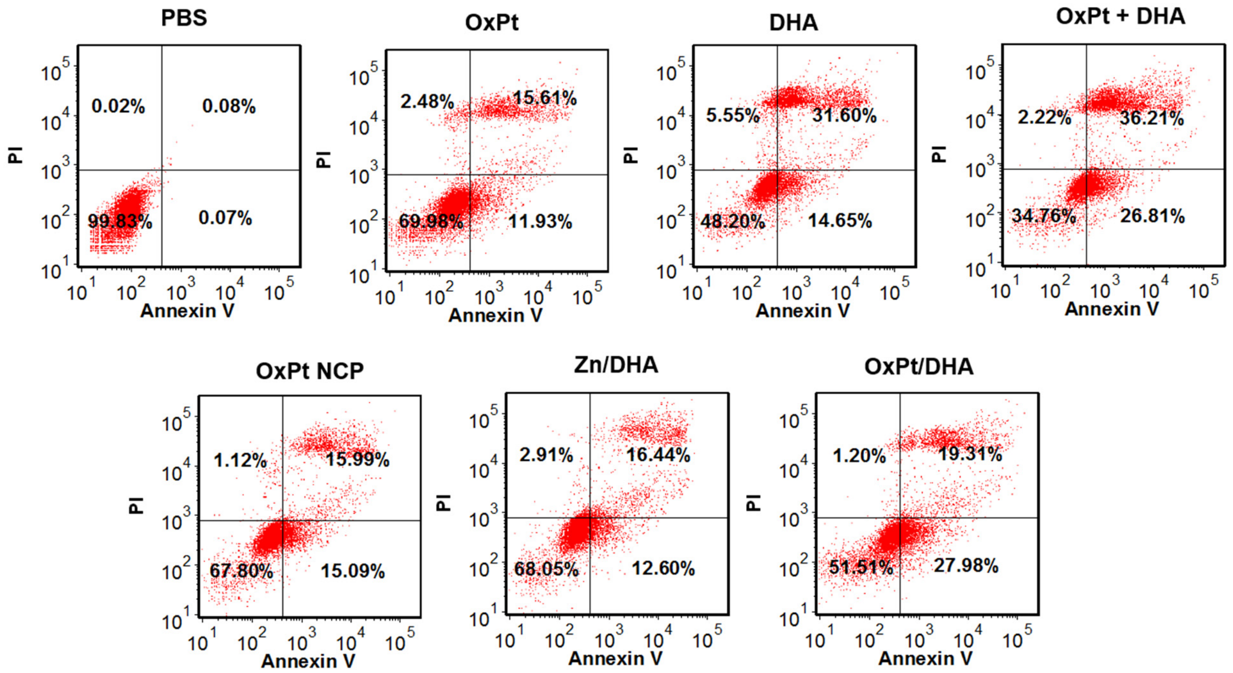

Supplementary Figure 15. Flow cytometry analysis for apoptosis of CT26 cells induced by OxPt, DHA or combinations.

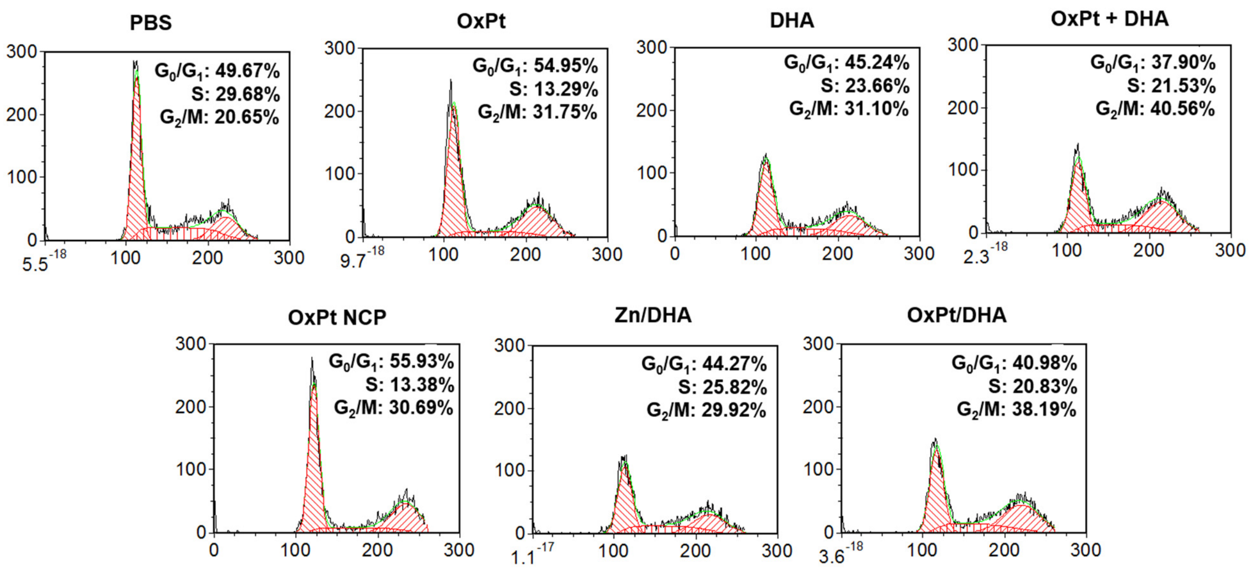

Supplementary Figure 16. Flow cytometry analysis for cell cycle change of CT26 cells induced by OxPt, DHA or combinations.

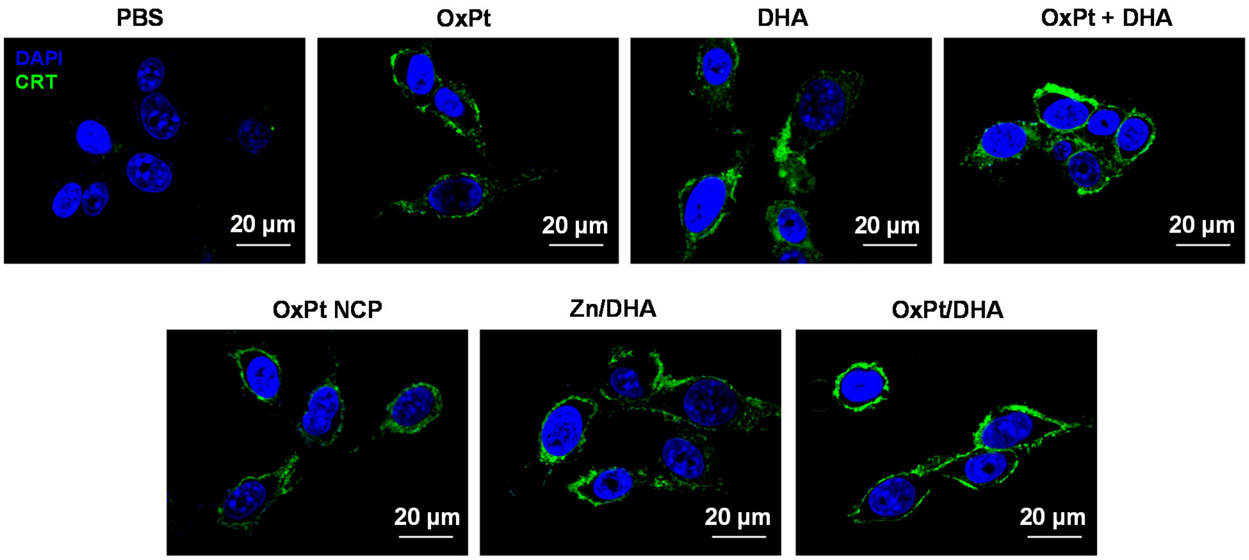

Supplementary Figure 17. Confocal images showing the CRT exposure on CT26 cells surface after treatment with OxPt, DHA or combinations.

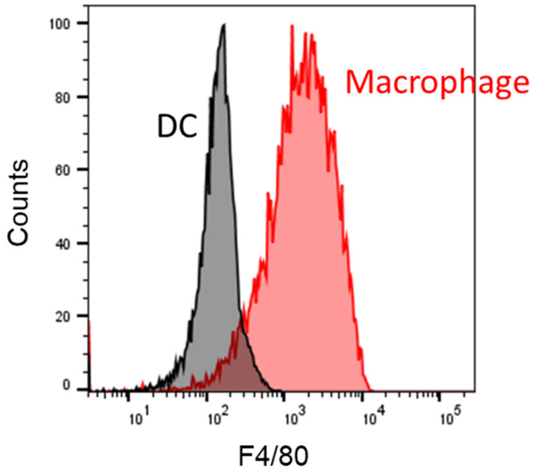

Supplementary Figure 18. The purities of bone marrow-derived dendritic cells ($CD11b^+CD11c^+$ $F4/80^-$) and macrophages ($CD11b^+$ $F4/80^+$).

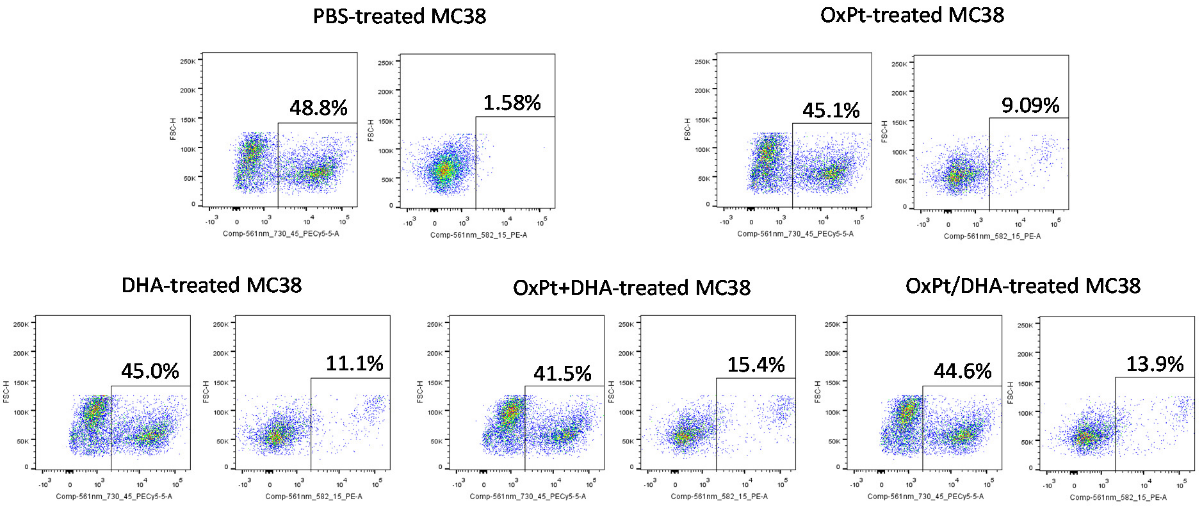

Supplementary Figure 19. Uptake of treated MC38 cells by bone marrow-derived dendritic cells.

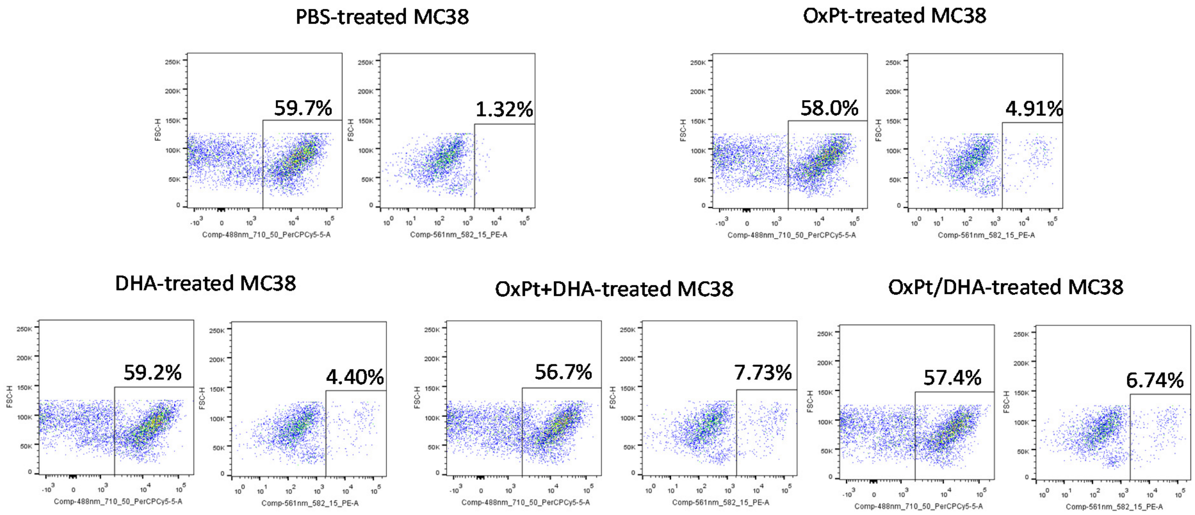

Supplementary Figure 20. Uptake of treated MC38 cells by bone marrow-derived macrophages.

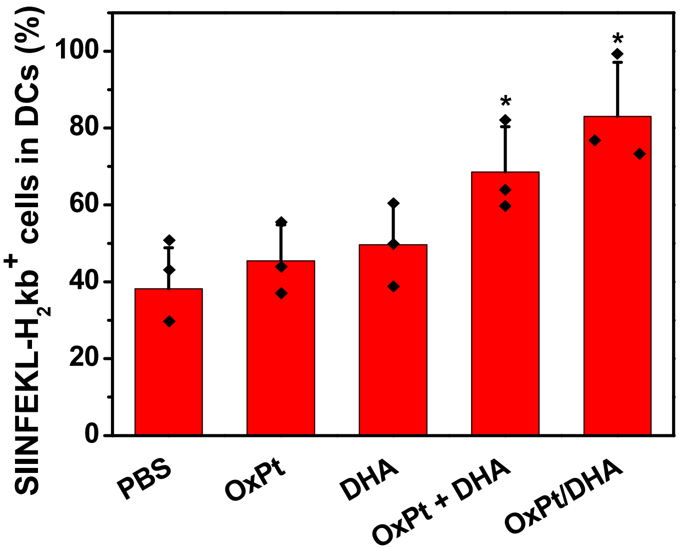

Supplementary Figure 21. Antigen presentation profiles of DCs exposed to OxPt/DHA-treated MC38-OVA cells. Data are expressed as means±SD.

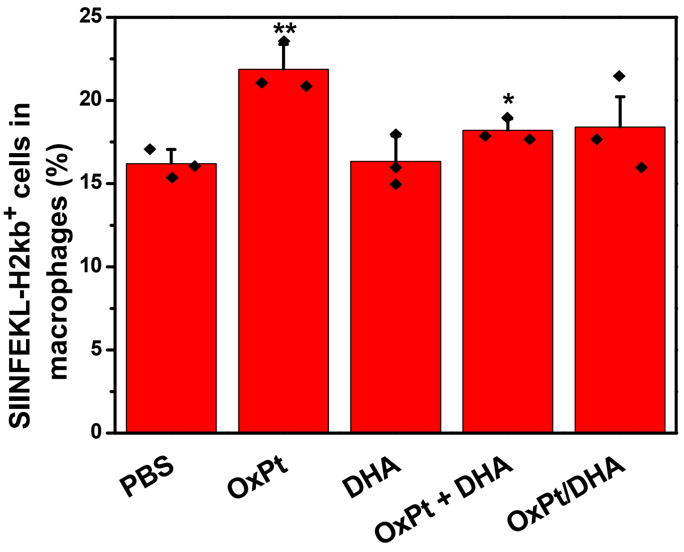

Supplementary Figure 22. Antigen presentation profiles of macrophages exposed to OxPt/DHA-treated MC38-OVA cells. Data are expressed as means±SD.

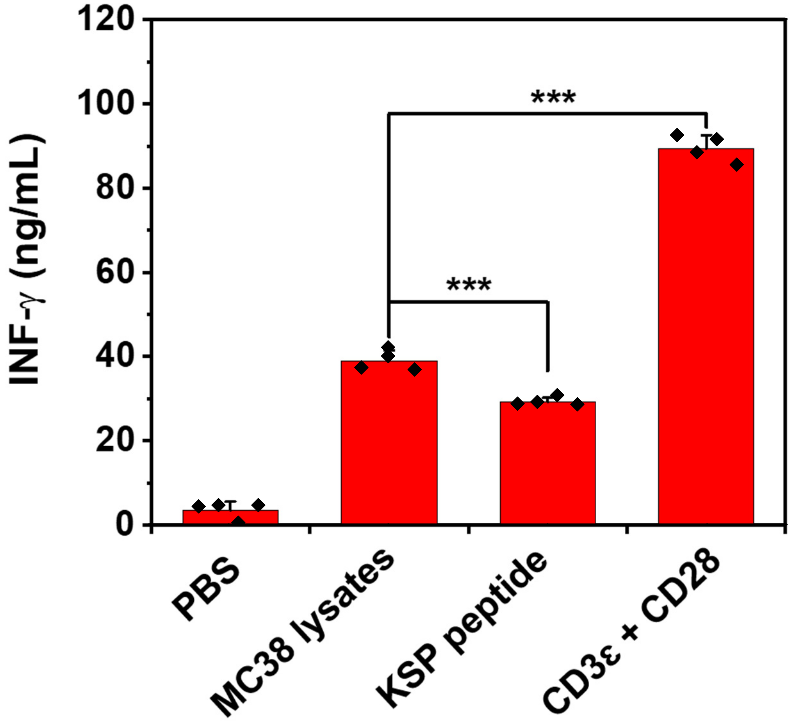

Supplementary Figure 23. Production of IFN-γ by draining lymph node cells after stimulation by OxPt/DHA-treated MC38 cell lysates, KSP peptide, and CD3ε plus CD28. Data are expressed as means±SD.

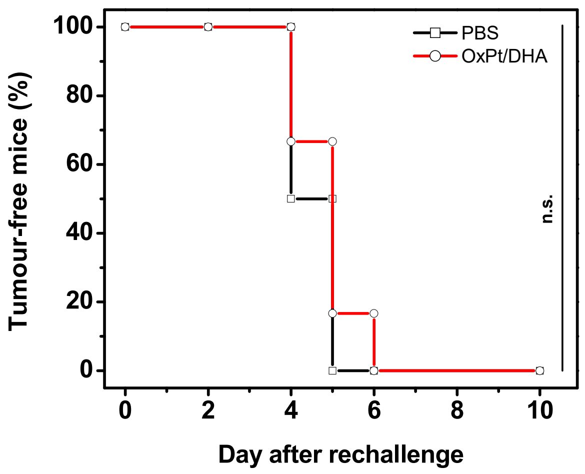

Supplementary Figure 24. Specific antitumour immunity elicited by OxPt/DHA-treated cells. Animals immunized with OxPt/DHA-treated MC38 cells that remain tumour-free 30 d after challenge with live MC38 were rechallenged with unrelated LL/2 cells.

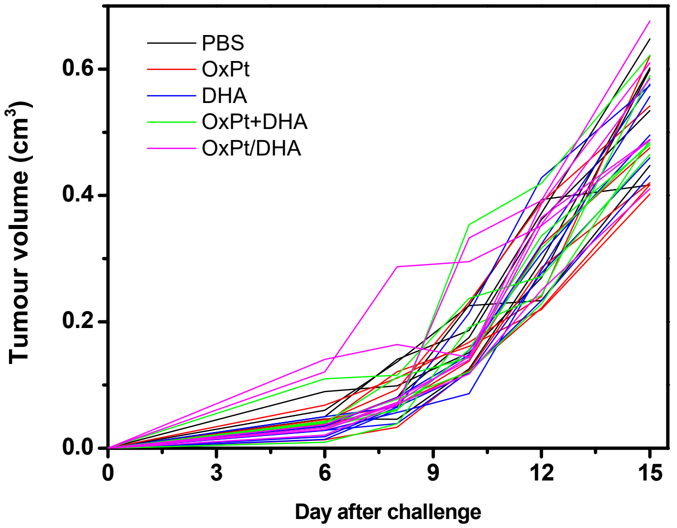

Supplementary Figure 25. Growth curves of challenged MC38 tumours on $Rag^{2-/-}$ mice.

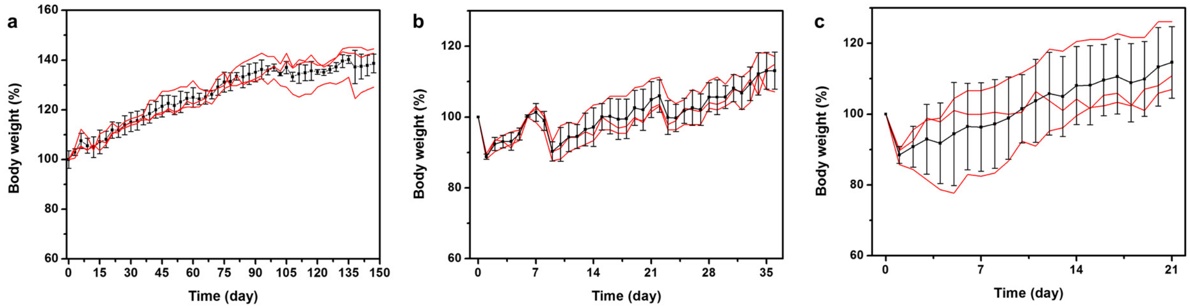

Supplementary Figure 26. Limited toxicity by chemotherapeutic NCPs. a, Body weight evolution of BALB/c mice after multiple injections (5 mg DHA/kg once every three days for a total of fifty doses). b, Body weight evolution of C57BL/6 mice after single injection (80 mg OxPt/kg) of OxPt/DHA. c, Body weight evolution of C57BL/6 mice after multiple injections (60 mg OxPt/kg once every week for a total of four doses) of OxPt/DHA. Data are expressed as means±SD (n=3).

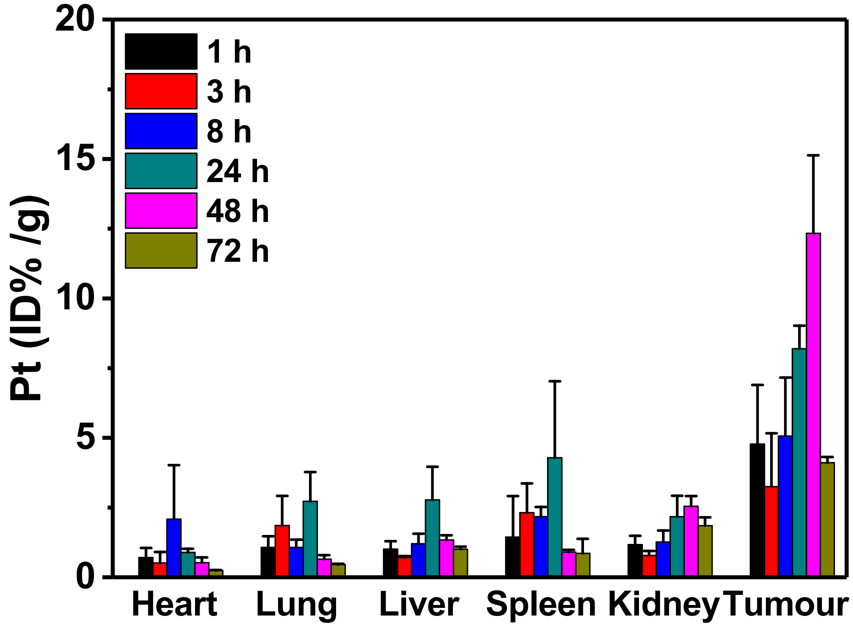

Supplementary Figure 27. Time-dependent Pt biodistribution of OxPt/DHA after i.p. injected to CT26-bearing mice. Data are expressed as means±SD.

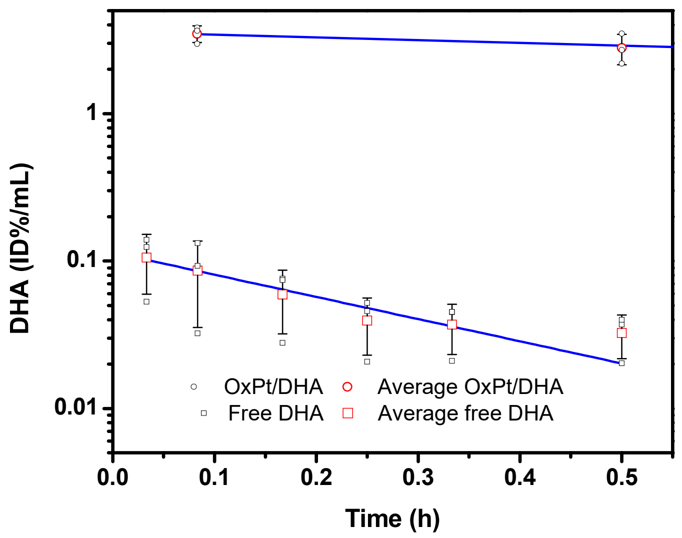

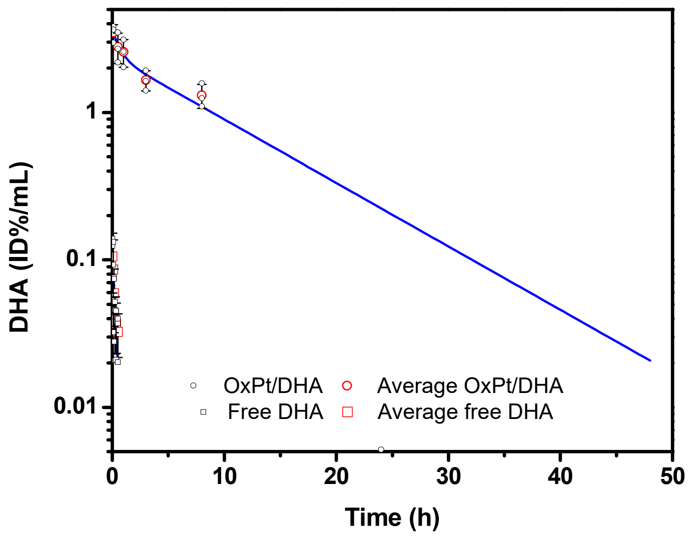

Supplementary Figure 28. Time-dependent DHA concentrations in the bloodstream from 0-0.5 h (top) and 0-48 h (bottom) after i.v. injection of DHA or OxPt/DHA to SD/CD rats. Data are expressed as means±SD.

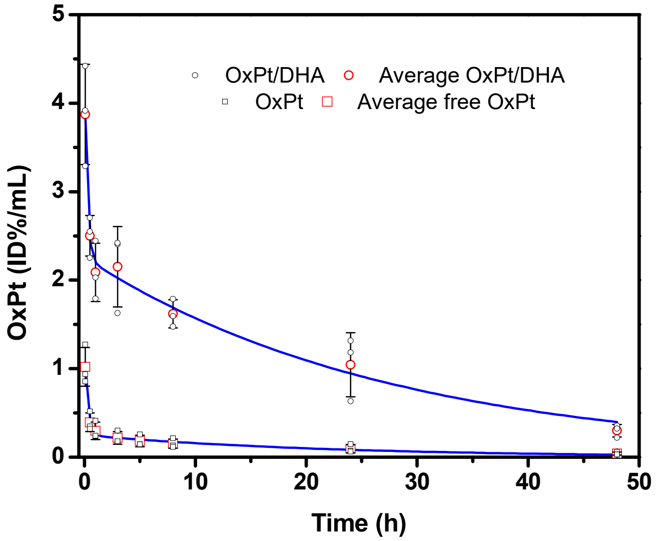

Supplementary Figure 29. Time-dependent OxPt concentrations in the bloodstream from 0-48 h after i.v. injection of DHA or OxPt/DHA to SD/CD rats. Data are expressed as means±SD.

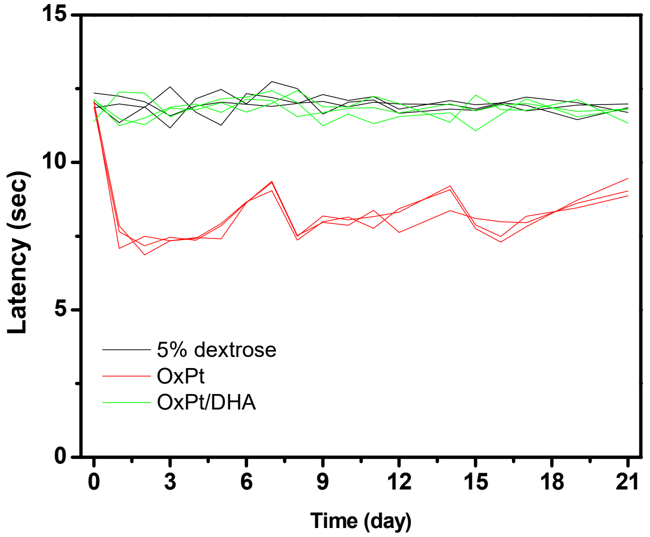

Supplementary Figure 30. Rats dosed with OxPt (8 mg/kg) showed thermal hyperalgesia compared to those treated with OxPt/DHA (8 mg OxPt/kg and 2.8 mg DHA/kg) or 5% dextrose control, causing the rats to withdraw their paws away from a heat stimulus after ~7-8 seconds as opposed to ~12 seconds.

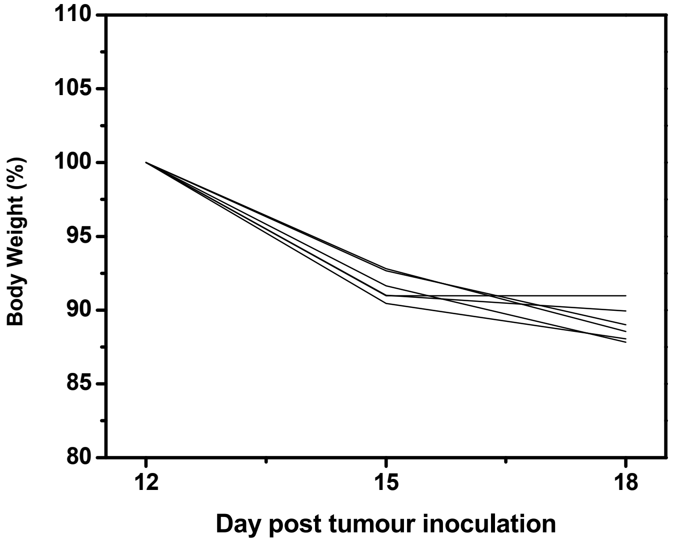

Supplementary Figure 31. Body weight evolution of mice treated with the free OxPt, DHA and anti-PD-L1 at 8 mg OxPt/kg, 2.8 mg DHA/kg and 75 μg/mouse anti-PD-L1.

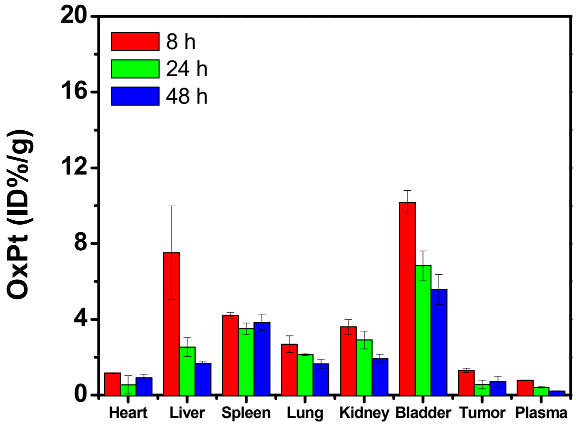

Supplementary Figure 32. Time-dependent Pt biodistribution of free OxPt after i.p. administration. Data are expressed as means±SD.

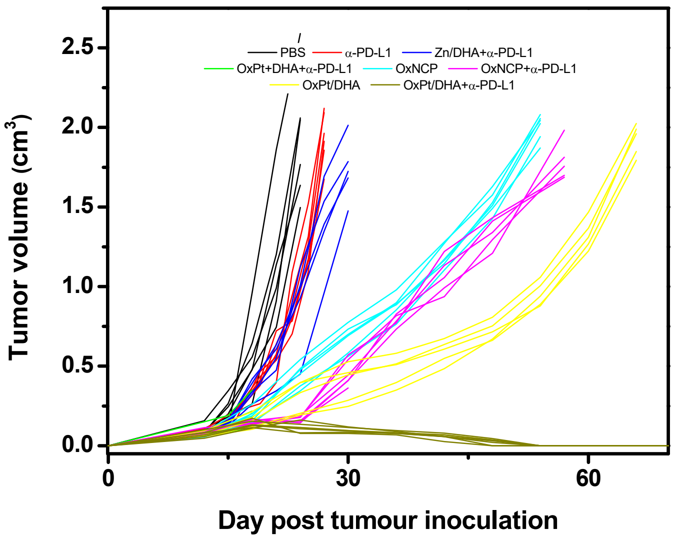

Supplementary Figure 33. CT26 tumour growth curve after treated with various formulations.

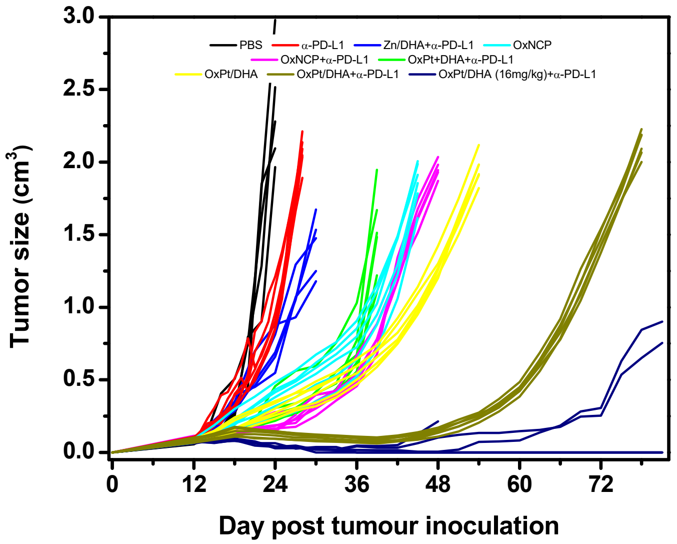

Supplementary Figure 34. MC38 tumour growth curve after treated with various formulations.

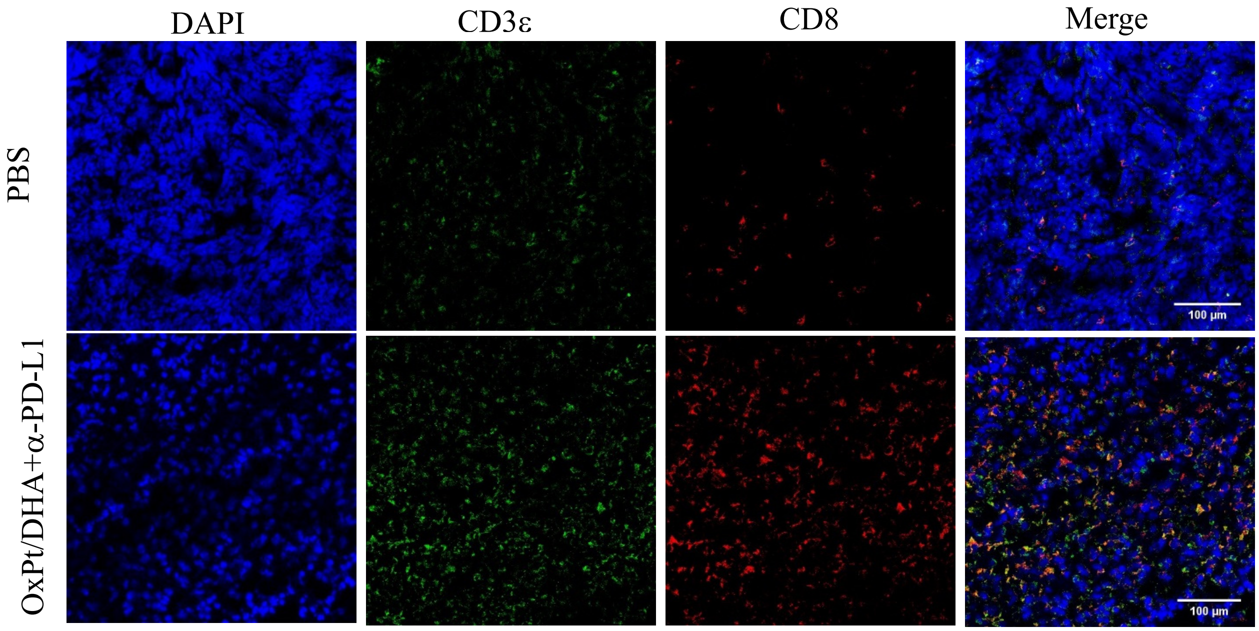

Supplementary Figure 35. $CD8^+$ T cell infiltration into the tumours of MC38 tumour-bearing mice 12 days after the first treatment.

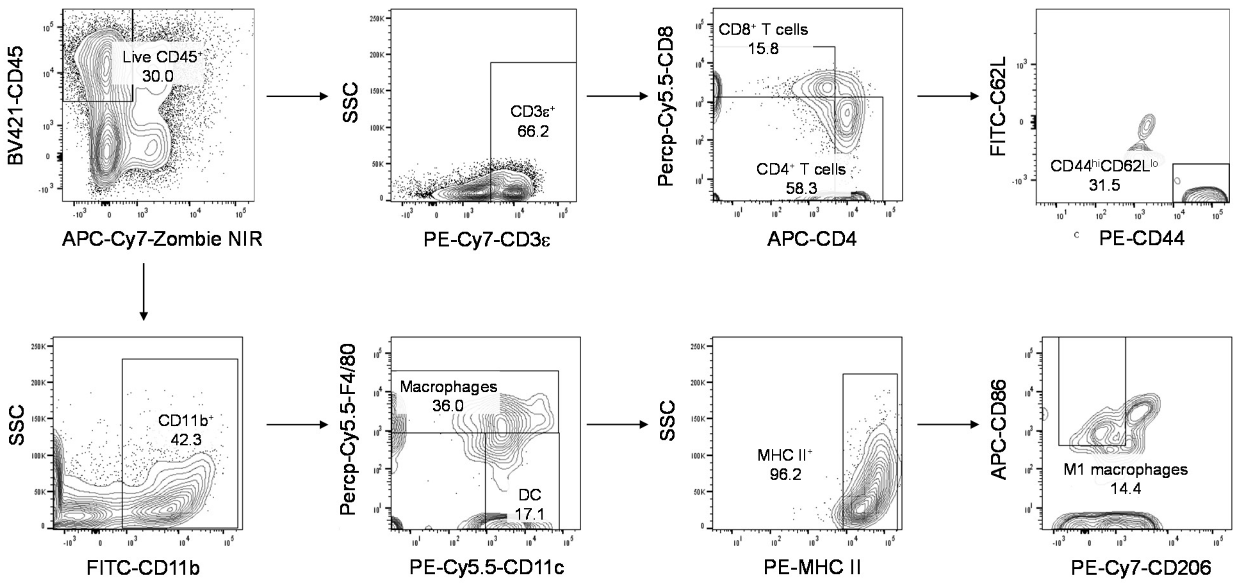

Supplementary Figure 36. Gating strategy of flow cytometry studies. All cells were gated from Zombie NIR negative cells. Total leukocytes were gated on $CD45^+$ cells; $CD4^+$ T cells were gated on $CD4^+CD3\varepsilon^+CD45^+$ cells; $CD8^+$ T cells were gated on $CD8\alpha^+CD3\varepsilon^+CD45^+$ cells; effector memory cells were gated on $CD44^{hi}CD62L^{lo}$ $CD8\alpha^+CD3\varepsilon^+CD45^+$ cells; macrophages were gated on $F4/80^+CD11b^+CD45^+$ cells; M1 macrophages were gated on $CD86^+CD206^-$ $MHCII^+F4/80^+CD11b^+CD45^+$ cells; dendritic cells were gated on $CD11c^+F4/80^-CD11b^+CD45^+$ cells.

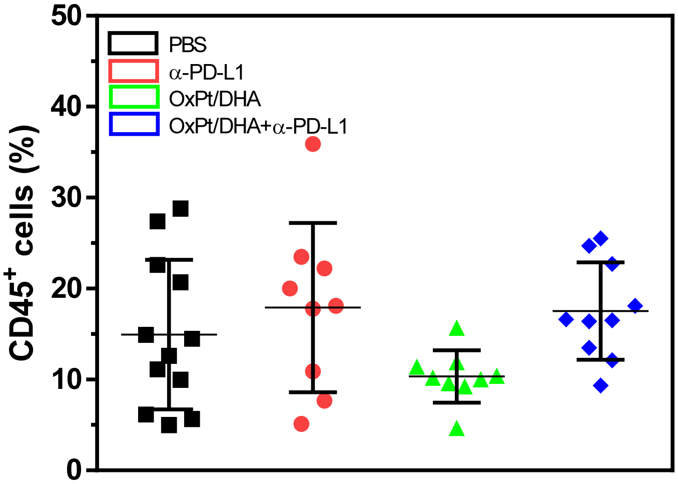

Supplementary Figure 37. CD45$^+$ leukocyte infiltration into tumours 12 days after the first treatment. Data are expressed as means±SD.

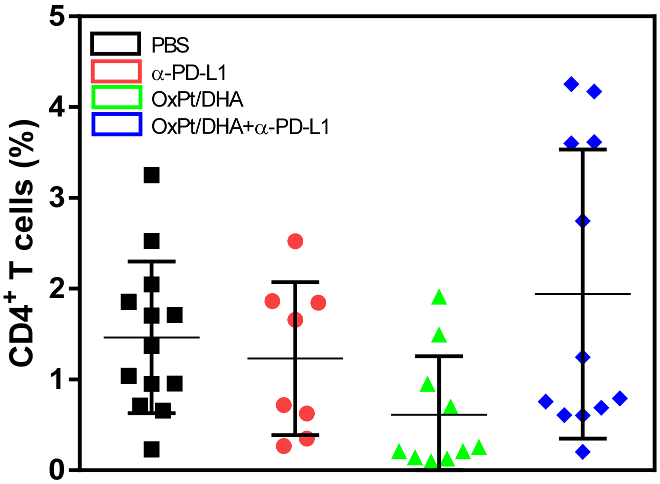

Supplementary Figure 38. CD4^{+} T cell infiltration into tumours 12 days after the first treatment. Data are expressed as means±SD.

Supplementary Table 1. OxPt and DHA IC_{50} values (μM).

	**OxPt**	**DHA**	**OxPt + DHA 1:0.5**	**OxPt + DHA 1:1**	**OxPt + DHA 1:2**
CT26 Free drug	9.1±0.7	2.6±0.6	3.2±0.4 (1.7±0.2) [a]	1.5±0.3 (1.5±0.3) [a]	1.1±0.3 (2.2±0.5) [a]
CT26 NCP	14.1±1.2	9.9±0.6	8.7±0.3 (4.3±0.2) [a]	6.8±0.7 (6.8±0.7) [a]	4.2±0.7 (8.3±1.3) [a]
MC38 Free drug	10.1±1.1	3.3±0.5	3.6±0.9 (1.8±0.5) [a]	1.6±0.2 (1.6±0.2) [a]	1.1±0.2 (2.3±0.3) [a]
MC38 NCP	15.6±1.5	7.4±0.6	8.9±0.4 (4.4±0.2) [a]	6.4±0.7 (6.4±0.7) [a]	4.4±0.6 (8.7±1.2) [a]

[a]The numbers in parentheses refer to DHA IC_{50} values.

Supplementary Table 2. Characterization of nanoparticles.

	Z-Average (d. nm)	PDI	Number (d. nm)	Loading	ε-potential (mV)
OxPt-bare	78.27 ± 0.18	0.159 ± 0.01	40.40 ± 3.08	28% OxPt, 16% Zn	
OxPt NCP	89.21 ± 1.17	0.136 ± 0.01	55.37 ± 2.28		-17.90 ± 0.46
OxPt/DHA	73.80 ± 0.36	0.174 ± 0.01	41.00 ± 0.62		-20.83 ± 1.25
Zn/DHA	98.60 ± 0.75	0.140 ± 0.01	63.50 ± 6.76		-23.85 ± 3.75

Supplementary Table 3. The effect of DHA loading on particle size.

	Z-Average (d. nm)	PDI	Intensity (d. nm)	Number (d. nm)
OxPt/DHA (1:0.5)	73.8 ± 0.36	0.174 ± 0.01	85.0 ± 4.23	41.0 ± 0.62
OxPt/DHA (1:1)	101.0 ± 2.37	0.159 ± 0.01	120.5 ± 1.36	60.8 ± 8.46
OxPt/DHA (1:2)	103.4 ± 1.01	0.116 ± 0.01	117.5 ± 1.66	69.7 ± 1.11

Supplementary Table 4. Crystallographic information of OxPt-bc.

**Name**	**OxPt-bc**
**Formula**	$C_{10}H_{10}N_4O_8Pt$
**Fw**	509.30
**Temperature (K)**	100
**Wavelength (Å)**	0.71073
**Crystal system**	Monoclinic
**Space group**	*C*2/*c*

***a*, Å**	10.9703(8)
***b*, Å**	14.5913(11)
***c*, Å**	10.2980(7)
**α, °**	90
**β, °**	118.233(2)
**γ, °**	90
***V*, Å^3**	1452.30(18)
Z	4
**Density (calcd. g/cm^3)**	2.329
**Absorption coeff. (mm^{-1})**	9.711
**F(000)**	960.0
**θ range data collection**	2.528 – 33.716
**Limiting indices**	-17<=h<=16<br>-22<=k<=22<br>-16<=l<=16
**Reflection collected**	26756
**Independent reflections**	2765
**R(int)**	0.0643
**Data/restraints/parameters**	2765/0/113
**Goodness-of-fit on *F*^{2}**	1.127
**Final R indices [I>2σ(I)]**	R1=0.0347, wR2=0.0715
**R indices (all data)**	R1=0.0483, wR2=0.0747

Reference

1. Duan, X., et al. Photodynamic Therapy Mediated by Nontoxic Core–Shell Nanoparticles Synergizes with Immune Checkpoint Blockade To Elicit Antitumor Immunity and Antimetastatic Effect on Breast Cancer. *J. Am. Chem. Soc.* 2016, **138**, 16686-16695.
2. Sheldrick, G. M. Crystal structure refinement with SHELXL. *Acta. Cryst.* **C71**, 3–8 (2015).
3. Dolomanov, O. V., Bourhis, L. J., Gildea, R. J., Howard, J. A. K., Puschmann, H. OLEX2: A complete structure solution, refinement and analysis program. *J. Appl. Crystallogr.* **42**, 339–341 (2009).
4. Sheldrick, G. M. A short history of SHELX. *Acta. Cryst.* **A64**, 112–122 (2008).
