# Supplementary material for: Immunostimulatory nanomedicines synergize with checkpoint blockade immunotherapy to eradicate colorectal tumors
Source: Nat Commun. 2019 Apr 23;10:1899. doi: 10.1038/s41467-019-09221-x (PMC6478897; doi:10.1038/s41467-019-09221-x)
Supplement: Supplementary file 2 — Reporting Summary [file 41467_2019_9221_MOESM2_ESM.pdf]

nature research

Corresponding author(s): Wenbin Lin

Last updated by author(s): Feb 26, 2019

Reporting Summary

Nature Research wishes to improve the reproducibility of the work that we publish. This form provides structure for consistency and transparency in reporting. For further information on Nature Research policies, see Authors & Referees and the Editorial Policy Checklist.

Statistics

For all statistical analyses, confirm that the following items are present in the figure legend, table legend, main text, or Methods section.

n/a	Confirmed	
☐	☒	The exact sample size (n) for each experimental group/condition, given as a discrete number and unit of measurement
☐	☒	A statement on whether measurements were taken from distinct samples or whether the same sample was measured repeatedly
☐	☒	The statistical test(s) used AND whether they are one- or two-sided<br>*Only common tests should be described solely by name; describe more complex techniques in the Methods section.*
☐	☒	A description of all covariates tested
☐	☒	A description of any assumptions or corrections, such as tests of normality and adjustment for multiple comparisons
☐	☒	A full description of the statistical parameters including central tendency (e.g. means) or other basic estimates (e.g. regression coefficient) AND variation (e.g. standard deviation) or associated estimates of uncertainty (e.g. confidence intervals)
☒	☐	For null hypothesis testing, the test statistic (e.g. F, t, r) with confidence intervals, effect sizes, degrees of freedom and P value noted<br>*Give P values as exact values whenever suitable.*
☒	☐	For Bayesian analysis, information on the choice of priors and Markov chain Monte Carlo settings
☒	☐	For hierarchical and complex designs, identification of the appropriate level for tests and full reporting of outcomes
☒	☐	Estimates of effect sizes (e.g. Cohen's d, Pearson's r), indicating how they were calculated

Our web collection on statistics for biologists contains articles on many of the points above.

Software and code

Policy information about availability of computer code

Data collection	Data was collected using Malvern Zetasizer Software, Agilent MassHunter, TopSpin, FACSDiva, Apex2, Fiji for CLSM, ELISA reader, ELISpot reader, crystal
Data analysis	Origin, Graphpad Prism, FlowJo, APEX, OLEX2, Agilent MassHunter

For manuscripts utilizing custom algorithms or software that are central to the research but not yet described in published literature, software must be made available to editors/reviewers. We strongly encourage code deposition in a community repository (e.g. GitHub). See the Nature Research guidelines for submitting code & software for further information.

Data

Policy information about availability of data

All manuscripts must include a data availability statement. This statement should provide the following information, where applicable:

- Accession codes, unique identifiers, or web links for publicly available datasets
- A list of figures that have associated raw data
- A description of any restrictions on data availability

Data Availability. The authors declare that all the data supporting the findings of this study are available within the article and its Supplementary Information files or from the corresponding author upon reasonable request. The crystal structure reported is deposited at the Cambridge Crystallographic Data Centre (CCDC) under deposition number CCDC 1875999. The crystallographic file can be obtained free of charge from the Cambridge Crystallographic Data Centre via http://www.ccdc.cam.ac.uk/data_request/cif.

Field-specific reporting

Please select the one below that is the best fit for your research. If you are not sure, read the appropriate sections before making your selection.

☒ Life sciences ☐ Behavioural & social sciences ☐ Ecological, evolutionary & environmental sciences

For a reference copy of the document with all sections, see nature.com/documents/nr-reporting-summary-flat.pdf

Life sciences study design

All studies must disclose on these points even when the disclosure is negative.

Sample size	Between groups, ANOVA was used to assess the difference on tumor inhibition of nanoparticles. Assuming the nanoparticles will decrease tumor weight to 25% of control, with proposed sample size, n=6/group, we will have a power of >80% to detect a significant difference at a significance level of 0.05.
Data exclusions	No data were excluded from analysis, except where mice were euthanized prior to the endpoint analysis for humanitarian reasons in accordance with our animal protocols.
Replication	In vitro experiments were completed in triplicate to successfully verify reproducibility. In vivo experiments were completed in duplicate or triplicate as noted by up to three people to successfully verify reproducibility and the results were pooled as noted.
Randomization	Animals were randomly distributed into treatment groups, such that the average starting tumor sizes across groups were approximately consistent.
Blinding	Data analysis was blinded for animal experiments.

Reporting for specific materials, systems and methods

We require information from authors about some types of materials, experimental systems and methods used in many studies. Here, indicate whether each material, system or method listed is relevant to your study. If you are not sure if a list item applies to your research, read the appropriate section before selecting a response.

Materials & experimental systems

n/a	Involved in the study	
☐	☒	Antibodies
☐	☒	Eukaryotic cell lines
☒	☐	Palaeontology
☐	☒	Animals and other organisms
☒	☐	Human research participants
☒	☐	Clinical data

Methods

n/a	Involved in the study	
☒	☐	ChIP-seq
☐	☒	Flow cytometry
☒	☐	MRI-based neuroimaging

Antibodies

Antibodies used

CD16/32 eBioscience REF# 14016186 clone 93 LOT# 1969562
CD45 BD Bioscience REF# 563890 clone 30-F11 LOT#8072537
CD3e eBioscience REF# 25003182 clone 145-2C11 LOT# 4304567
CD8 eBioscience REF# 45-0081-82 clone 53-6.7 LOT# 4291993
CD11b eBioscience REF# 11-0112-85 clone M1/70 LOT# 4319899
CD11c eBioscience REF# 35-0114-82 clone N418 LOT# 4303855
F4/80 eBioscience REF# 4504801-82 clone BM8 LOT# 4341619
MHC II eBioscience REF# 12-5320-82 clone AF6-120.1 LOT# E19826-102
CD86 BD Bioscience REF# 553691 clone GL1 LOT# 3217925
CD206 Biolegend REF# 141719 clone C068C2 LOT# B239603
CD44 eBioscience REF# 12-0441-82 clone IM7 LOT# 4277534
CD62L Biolegend REF# 104405 clone MEL-14 LOT# B258725
Gr-1 eBioscience REF#17-5931-82 clone RB6-8C5 LOT#4303113
SINFEKL/H-K2B Biolegend 141603 25-D1.16 B231195
Cytochrome C eBioscience 11-6601-82 6H2 4287755
Calreticulin Enzo ADI-SPA-601-488-F FMC57 3011604
PD-L1 BioXCell BE0101 10F.9G2 5786/1215

Validation

All antibodies were validated by the manufacturers.

October 2018

Eukaryotic cell lines

Policy information about cell lines

Cell line source(s)	CT26: ATCC<br>MC38: ATCC<br>LL/2: ATCC<br>4T1: ATCC
Authentication	The cells were not authenticated before use.
Mycoplasma contamination	The cells tested negative for mycoplasma contamination according to the MycoAlert detection kit (Lonza Nottingham, Ltd.).
Commonly misidentified lines<br>(See ICLAC register)	None of these cell lines are used.

Animals and other organisms

Policy information about studies involving animals; ARRIVE guidelines recommended for reporting animal research

Laboratory animals	Male and female BALB/c and C57BL/6 mice aged 6-8 weeks and female SD/CD rats aged 6-8 weeks were used.
Wild animals	No wild animals were used.
Field-collected samples	No samples were collected in the field.
Ethics oversight	The IACUC at the University of Chicago approved our study protocol.

Note that full information on the approval of the study protocol must also be provided in the manuscript.

Flow Cytometry

Plots

Confirm that:

☒ The axis labels state the marker and fluorochrome used (e.g. CD4-FITC).

☒ The axis scales are clearly visible. Include numbers along axes only for bottom left plot of group (a 'group' is an analysis of identical markers).

☒ All plots are contour plots with outliers or pseudocolor plots.

☒ A numerical value for number of cells or percentage (with statistics) is provided.

Methodology

Sample preparation	Cells grown in culture were trypsinized, washed twice with PBS, and then fixed as necessary and stained with antibodies. Live cell suspensions were obtained by excising animal organs, grinding the tissues, washing the cells with PBS, and then staining as necessary with antibodies.
Instrument	FACS LSR Fortessa
Software	BD FACSDiva was used to collect data. FlowJo and FCS Express were used to analyze data.
Cell population abundance	No post-sort fractions were collected.
Gating strategy	All experiments included an unstained sample and single-stained samples as compensation to define negative and positive cell populations. The preliminary FSC/SSC gates excluded cell fragments and then gated on yellow, Zombie NIR, or PI to exclude dead cells as appropriate. Positive and negative cells were gated based on the unstained or single-stained samples. Total leukocytes were gated on CD45+ cells. CD4+ T cells were gated on CD4+CD3e+CD45+ cells. CD8+ T cells were gated on CD8a+CD3e+CD45+. Effector memory cells were gated on CD44hi CD62Llo CD8a+CD3e+CD45+ cells. Macrophages were gated on F4/80+CD11b +CD45+ cells. M1 macrophages were gated on CD86+CD206-MHCII+F4/80+CD11b+CD45+ cells. Dendritic cells were gated on CD11c+F4/80-CD11b+CD45+ cells.

☒ Tick this box to confirm that a figure exemplifying the gating strategy is provided in the Supplementary Information.
